# A Multi-Center Validated Subtyping Model of Esophageal Cancer Based on Three Metabolism-Related Genes

**DOI:** 10.3389/fonc.2021.772145

**Published:** 2021-10-25

**Authors:** Yu Liu, Liyu Wang, Lingling Fang, Hengchang Liu, He Tian, Yujia Zheng, Tao Fan, Chunxiang Li, Jie He

**Affiliations:** ^1^ Department of Thoracic Surgery, National Cancer Center/National Clinical Research Center for Cancer/Cancer Hospital, Chinese Academy of Medical Sciences and Peking Union Medical College, Beijing, China; ^2^ Department of Colorectal Surgery, National Cancer Center/National Clinical Research Center for Cancer/Cancer Hospital, Chinese Academy of Medical Sciences and Peking Union Medical College, Beijing, China

**Keywords:** esophageal squamous cell carcinoma, immune infiltration, metabolism, prognosis, bioinformatic

## Abstract

Metabolic reprogramming is a hallmark of malignancy. Understanding the characteristics of metabolic reprogramming in esophageal squamous cell carcinoma (ESCC) helps uncover novel targets for cancer progression. In this study, 880 metabolism-related genes were identified from microarray data and then filtered to divide patients into two subgroups using consensus clustering, which exhibits significantly different overall survival. After a differential analysis between two subtypes, 3 genes were screened out to construct a two subtypes decision model on the training cohort (GSE53624), defined as high-risk and low-risk subtypes. These risk models were then verified in two public databases (GSE53622 and TCGA-ESCC), an independent cohort of 49 ESCC patients by RT-qPCR and an external cohort of 95 ESCC patients by immunohistochemistry analysis (IHC). Furthermore, the immune cell infiltration of regulatory T cells (Tregs) and plasma cells showed a significant difference between the high and low-risk subtypes in the IHC experiment with 119 ESCC patients. In conclusion, our study indicated that three metabolism-related prognostic genes could stratify patients into subgroups and were associated with immune infiltration, clinical features and clinical outcomes.

## Introduction

Esophageal cancer is a common malignant tumor worldwide. China has the highest incidence of esophageal cancer in the world, and more than 90% of cases are esophageal squamous cell cancer (ESCC), which has high degree of malignancy and a poor prognosis (1). Metabolic reprogramming is a hallmark of cancer (2). Several factors might have an effect on tumor metabolism. Intrinsic factors include the characteristics of the parental tissue and new properties arising in the tumor cells. Extrinsic factors including the tumor microenvironment and the metabolic state of the patients, such as obesity, diabetes and other metabolic disorders, could all contribute to metabolic phenotypes in tumors. Tumor progression always involves metabolic needs, and vulnerabilities arise. The early stages of tumor growth require nutrient uptake and biosynthesis, but with the progression of tumors, such as metastasis and therapy resistance, metabolic needs change to resist oxidative stress and enhance the level of oxidative phosphorylation (3). Hence, it is necessary to characterize ESCC progression and explore prognosis-related genes from a metabolic perspective.

The development of omics techniques makes the selection of metabolism-related diagnostic or prognostic markers more effective. A recent study identified a novel plasma diagnostic biomarker panel consisting of eight metabolic molecules that could clearly distinguish ESCC patients from controls through untargeted metabolomics analysis (4). A four-gene metabolic signature could effectively predict the overall survival (OS) of patients with hepatocellular cancer (5). Glycolysis is a main metabolic pathway and is characteristically altered in cancer cells, and a glucose metabolism gene-related prognostic signature built for glioblastoma has the ability to distinguish the clinical and molecular features of the disease (6). Karasinska et al. demonstrated that glycolytic and cholesterogenic gene expression profiles could identify distinct subgroups associated with differences in survival and known prognostic pancreatic tumor subtypes and help develop personalized therapies targeting unique tumor metabolic profiles (7). These studies all indicated that metabolism-related markers have good clinical application value.

In our study, we found that metabolism-related genes associated with prognosis could stratify ESCC patients into two subgroups and were associated with tumor grade and OS. Then, we established a metabolism-related prognostic signature and showed a better predictive ability for OS. Further analysis showed that the prognostic signature was associated with several tumor progression-related pathways and with infiltration of several immune cell types. These results showed that the metabolism-related signature serves as a prognostic marker and has clear clinical application value.

## Materials and Methods

### Patient Samples

For the microarray study, ESCC tissues from 179 patients from Gene Expression Omnibus (GEO) database and 80 patients’ clinical data from the Cancer Genome Atlas (TCGA) were used. Forty-nine ESCC patient tissue samples were collected and patients underwent surgical treatment in National Cancer Center/Cancer Hospital of Chinese Academy of Medical Sciences from January 2012 to December 2012. For the immunohistochemistry (IHC) study, an ESCC tissue array with 95 patients who underwent surgery between January 2009 and December 2010 was obtained from Xinchao (Shanghai, China). Moreover, 119 ESCC patient tissues from microarrays were collected for IHC analysis. All patients were confirmed to have primary ESCC after surgery, and our study was approved by the Medical Ethics Committee of the Cancer Institute and Hospital, Chinese Academy of Medical Sciences.

### Data Collection

The expression profiles of GSE53624 and GSE53622 were downloaded from GEO database (http://www.ncbi.nlm.nih.gov/geo) (8, 9). In addition, the transcriptome expression profiles of ESCC and multitype tumors were acquired from the TCGA (https://tcga-data.nci.nih.gov/). The fragments per kilobase of transcript per million mapped reads (FPKM) data and clinical data were used and downloaded.

### Consensus Clustering and Construction of a Prognostic Signature

The metabolism-related gene set was selected from the Molecular Signature Database (MSigDB) Kyoto Encyclopedia of Genes and Genomes (KEGG) gene sets (10). We used the keywords “Metabolism” and “Metabolic” to screen out metabolism-related KEGG pathways and acquired metabolism-related genes in GSE53624 and GSE52622. A total of 880 metabolism-related genes were collected in our studies, and the gene list is shown in **Supplementary Table 1**. Univariate Cox proportional hazards regression (PHR) analysis and Kaplan-Meier survival analysis were used to select prognosis-related genes associated with OS from the metabolism-related genes. Moreover, consensus clustering was performed on prognosis-related genes using ConsensusClusterPlus v1.38 (11). The best classification was selected according to the consensus cumulative distribution function (CDF) and delta area.

Univariate and multivariate stepwise Cox PHR analyses were used to construct a metabolism-related prognosis mRNA signature based on OS (GSE53624). Starting with the gene with the largest univariate z-score, we gradually added one gene that showed an association with OS and evaluated the prognostic performance at each step. The process was repeated until no improvement was found. The best risk score cutoff value was selected, and Kaplan-Meier survival curve analysis was used to calculate differences between groups. In addition, multivariate Cox regression analysis was applied to determine the independent prognostic ability of our signature and clinical factors.

Two public databases (GSE53622 and TCGA-ESCC) were used to verify our prognosis signature, and IHC analysis was used to verify the protein expression levels in ESCC tissue. Subsequently, the clinical predictive ability of the signature combined with TNM stage and tumor grade was analyzed. Time-dependent receiver operating characteristic (ROC) curves and area under the curve (AUC) values were analyzed to determine the predictive power. Finally, another type of tumor from the TCGA was assessed to explore the application value of our prognostic signature.

### Immune Infiltration and Functional Enrichment Analysis

The Tumor Immune Estimation Resource (TIMER2.0; http://timer.cistrome.org/) was used to estimate immune infiltration with the CIBERSORT method (12). We analyzed the relationship between immune infiltration and our prognostic signature and verified it by IHC analysis.

Gene Ontology (GO) and KEGG enrichment analyses of metabolism-related prognostic genes were performed with DAVID (https://david.ncifcrf.gov/) (13). The cutoff risk score was used to stratify samples into a high- or low-risk group criterion. GSEA analyses were used to explore hallmark pathway enrichment in the high- and low-risk groups. The hallmark gene set (h.all.v7.1.symbols) was selected for use in the cohort and validation set.

### RNA Isolation and RT-qPCR

Total RNA from ESCC tissues was isolated using the TRIzol protocol (Thermo). cDNA synthesis was performed using the EasyScript^®^ All-in-one First step cDNA Synthesis SuperMix for qPCR (AE341, TransGen). qPCR was performed using the PerfectStart™ Green qPCR SuperMix (AQ602, TransGen) on an ABI 7900HT Real-Time PCR Thermocycler (Life Technologies). The gene expression data were normalized to the results of the endogenous control β-actin. The delta CT method was used, and all samples were analyzed in triplicate. The primer sequences are shown in **Supplementary Table 2**.

### Immunohistochemistry Analysis

For validation of prognostic genes, ESCC patient tissue arrays containing 95 samples were used to analyze prognosis and gene expression differences. FOXP3 was a specific marker of Treg cells. We selected the 119 ESCC tissues with high expression of FOXP3 that could be matched to samples in the GSE53624 dataset, to explore the relationship between immune infiltration and gene expression. IHC staining was performed according to the manufacturer’s instructions. Antibodies against CD38 (Abcam, #ab108403), INPP5E (Abcam, #ab236108), POLR3G (Abcam, #ab230854) and FOXP3 (CST, #98377) were used in our study. The cytoplasmic staining score was calculated by the intensity score × percentage score, the nuclear staining score was calculated by the percentage (range from 0-3 score). Immune cell staining was calculated as the percentage of field area. Independent pathologists were given an IHC score or percentage.

### Statistical Analysis

Clinical statistical analysis was tested by Student’s *t* test for two groups, one-way ANOVA for three or more groups, and chi-squared tests or Wilcoxon tests for cohort clinicopathologic features or differential expression. A *p*-value < 0.05 was considered statistically significant. R software and several packages, including ConsensusClusterPlus, ggplot2, limma, survival, survminer and timeROC (bioconductor.org/biocLite.R), were used for the statistical analysis and plotting. SPSS version 25 software and GraphPad Prism were also used.

## Results

### Metabolism-Related Prognostic Gene Expression Identifies Two Distinct Subgroups of ESCC

**Figure 1:**
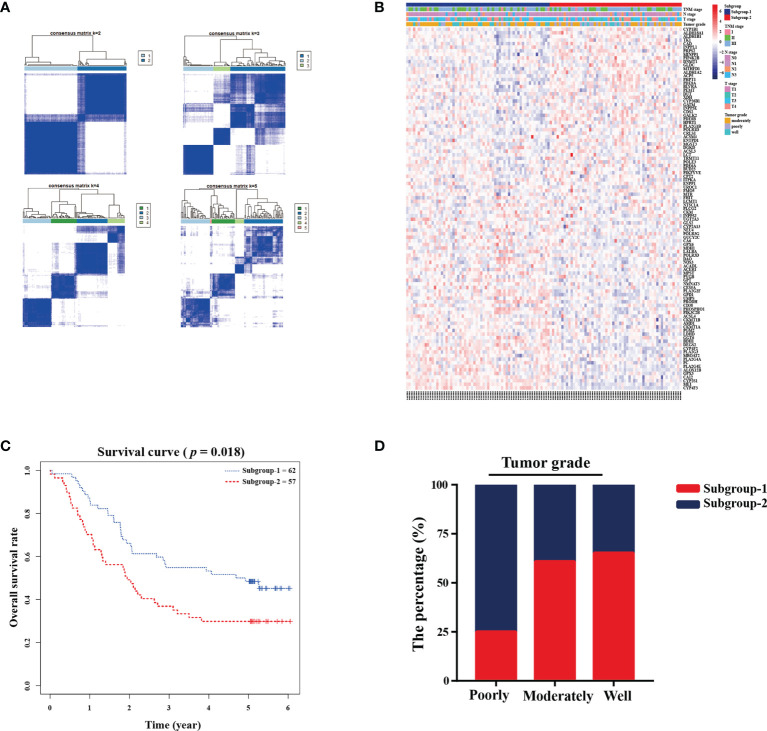
Metabolic-related prognosis gene sets were associated with tumor grade and overall survival. **(A)** Consensus clustering matrix of 119 ESCC samples for k=2 to k=5. **(B)** Heatmap and clinicopathologic features of the two subgroups defined by the metabolic-related gene sets. **(C)** Kaplan-Meier survival analysis for the two subgroups. **(D)** The percentage of two subgroups in tumor grade.

A total of 880 metabolism-related genes were filtered from the mRNA microarray data (GSE53624 and GSE53622). A total of 101 prognosis-related genes met our criteria and were used to identify subgroups. The cluster stability increased from k = 2 to k = 10 (**Figure 1A** and **Supplementary Figure S1**). We found that consensus clustering could distinguish the clinical characteristics into two subgroups (k = 2) in 119 patients (**Figure 1B**). There was a significant difference between subgroup-1 and subgroup-2 (*p* = 0.018; **Figure 1C**). Further analysis showed that subgroup 2 was closely correlated with poor grade (75%, *p* = 0.001; **Figure 1D**). The DAVID functional enrichment results also showed that the 101 prognosis-related genes could be enriched in several metabolic-related pathways (**Supplementary Figure S2**). These results indicated that these metabolism-related genes might be associated with tumor prognosis and involved in tumor progression.

### Identification of a Three-Gene Metabolism-Related Prognostic Signature in Patients With ESCC

**Figure 2:**
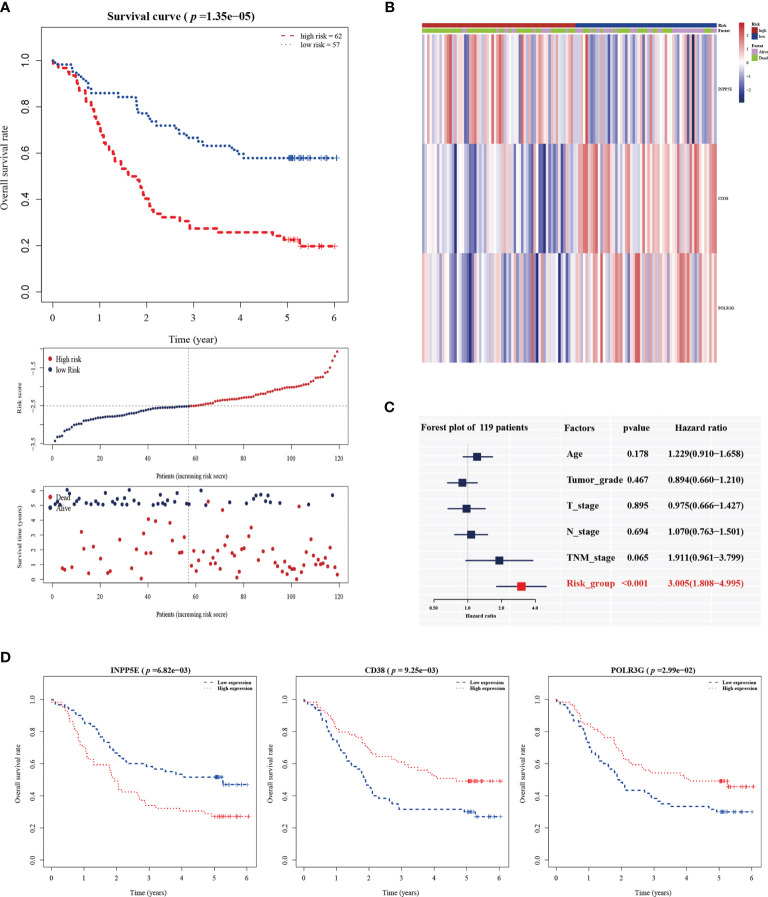
The establishment of a metabolic-gene prognosis signature in the training group. **(A)** Kaplan-Meier survival analysis of overall survival, the distribution of patients’ risk scores, and survival status for the high- and low-risk groups. **(B)** The heatmap of three gene signatures. **(C)** The independent prognostic factor of the three gene signature by multivariate Cox regression analysis. **(D)** Kaplan-Meier survival analysis for INPP5E, CD38 and POLR3G expression.

The 101 prognosis-related and, 135 significantly differentially expressed metabolism-related genes (logFC > 1; adjusted *p* < 0.01) between subgroup 1 and subgroup 2 were selected to calculate estimated regression coefficients by multivariable stepwise Cox PHR analysis. The three-gene prognostic signature was established in 119 patients (GSE53624) with ESCC, and we calculated the risk score and divided patients into high-risk (n=62) and low-risk (n=57) groups with the best cutoff point of -2.513. There was a significant difference between the high- and low-risk groups in terms of OS (*p* < 0.001, **Figure 2A**). The heatmap also showed that the expression of the three genes in the signature was significantly different in the high- and low-risk groups (**Figure 2B**). In addition, multivariate Cox analysis demonstrated that our three-gene signature could serve as an independent predictive factor for poor OS (HR =3.005; 95% CI: 1.80-4.99; *p*<0.001; **Figure 2C**). Finally, the metabolism-related prognosis signature (CD38, INPP5E and POLR3G; **Figure 2D**) with the best prognostic performance was selected. The estimated regression coefficients were identified as follows: risk score = (-0.14797 × expression level of CD38) + (0.03732 × expression level of INPP5E) +(-0.21290 × expression level of POLR3G).

### Validation of the Prognostic Value of the Metabolism-Related Prognostic Signature

**Figure 3:**
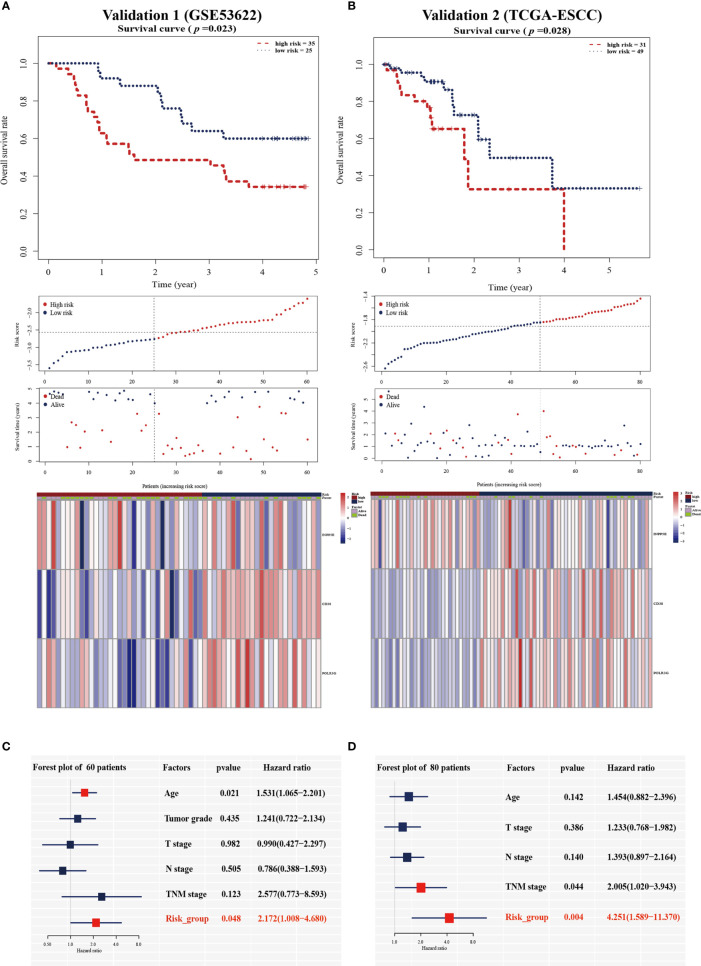
Validation of the prognostic signature in the two validation cohorts. Kaplan-Meier survival analysis of overall survival, the distribution of patients’ risk scores, survival status, and heatmap of the three gene signatures in the validation cohort of 60 patients **(A)** and 80 patients **(B)**. Multivariate Cox regression analysis in the two validation cohorts **(C, D)**.

**Figure 4:**
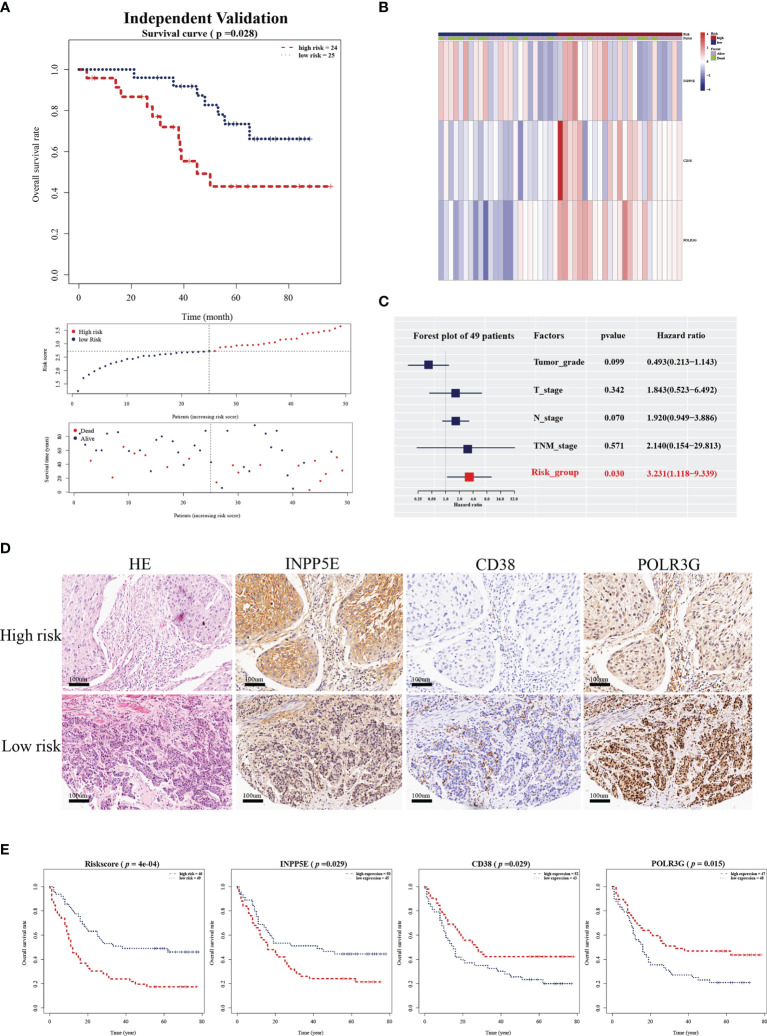
Validation of the independent cohort by RT-qPCR and IHC analysis. **(A)** Kaplan-Meier survival analysis of overall survival, the distribution of patients’ risk scores, and survival status for the high- and low-risk group in an independent validation cohort with 49 ESCC patients. **(B)** Heatmap of three gene signatures in an independent validation cohort with 49 ESCC patients. **(C)** The independent prognostic factor of the three gene signature by multivariate Cox regression analysis in an independent validation cohort with 49 ESCC patients. **(D)** HE staining and INPP5E, CD38 and POLR3G expression were analyzed by IHC analysis in an external validation cohort of 95 ESCC patients. **(E)** Kaplan-Meier survival analysis of overall survival for the risk score, INPP5E, CD38 and POLR3G in the IHC validation cohort.

To validate the prognostic value in public datasets, we selected 60 samples from the GSE53622 dataset and 80 samples from the TCGA dataset, and the same estimated regression coefficients and best cutoff values were selected. We found that our signature could effectively distinguish the high- and low-risk groups in the GSE53622 (*p*=0.023, **Figure 3A**) and TCGA (*p*=0.028, **Figure 3B**) datasets. Multivariate Cox analysis also demonstrated that our signature could serve as an independent predictive factor for OS in the GSE53622 (HR =2.172; 95% CI: 1.00-4.68; *p*=0.048; **Figure 3C**) and TCGA (HR =4.251; 95% CI: 1.589-11.37; *p*=0.004; **Figure 3D**) datasets. RT-qPCR was used to analyze an independent cohort of 49 ESCC patients and showed good predictive ability (**Figures 4A‒C**). The detailed clinical information is shown in **Table 1**.

**Table 1:** The clinicopathological characteristics stratified by prognosis signature in 49 ESCC patients.

Variables	Low risk		High risk	*p*-value
No. of patients	25	24		
Age				0.111
<60	14 (56.00%)	8 (33.33%)		
≥60	11 (44.00%)	16 (66.67%)		
Gender				0.889
Female	12 (48.00%)	12 (50.00%)		
Male	13 (52.00%)	12 (50.00%)		
Smoking				0.458
Yes	12 (48.00%)	9 (37.50%)		
No	13 (52.00%)	15 (62.50%)		
Drinking				0.684
Yes	9 (36.00%)	10 (41.67%)		
No	16 (64.00%)	14 (58.33%)		
Tumor grade				0.158
Well	5 (20.00%)	2 (8.33%)		
Moderately	11 (44.00%)	17 (70.83%)		
Poorly	9 (36.00%)	5 (20.83%)		
T stage				0.958
T1	2 (8.00%)	3 (12.50%)		
T2	5 (20.00%)	5 (20.83%)		
T3	16 (64.00%)	14 (58.33%)		
T4	2 (8.00%)	2 (8.33%)		
N stage				0.447
N0	9 (36.00%)	7 (29.16%)		
N1	5 (20.00%)	9 (37.50%)		
N2	6 (24.00%)	6 (25.00%)		
N3	5 (20.00%)	2 (8.33%)		
TNM stage				0.725
I	1 (4.00%)	2 (8.33%)		
II	7 (28.00%)	8 (33.33%)		
III	17 (68.00%)	14 (58.33%)		
Fustat				0.196
Alive	18 (72.00%)	13 (54.16%)		
Death	7 (28.00%)	11 (45.83%)		
OS	59.864 ± 17.933	42.079 ± 27.048		0.009**

**p < 0.01.

We next further validated the prognostic value by IHC analysis. The ESCC tissue array including 95 patients was used as an external validation cohort and divided into two groups based on the IHC score and area percentage in the ESCC slides (**Figure 4D**). The detailed clinical information is shown in **Table 2**. The risk scores were also calculated with the same estimated regression coefficients based on the IHC scores of POLR3G and INPP5E and the area percentage of CD38. We also found a significant difference in risk score between the high- and low-risk groups (**Figure 4E**). Consistently, CD38, INPP5E and POLR3G were also significantly associated with OS by IHC analysis (**Figure 4E**).

**Table 2:** The clinical characteristics at baseline, stratified by risk score in IHC validation group.

Variable	Low risk	High risk	*p*-value
No. of patients	49	46	
Age			0.866
<60	11 (22.45%)	11 (23.91%)	
≥60	38 (77.55%)	35 (76.09%)	
Gender			0.052
Male	36 (73.47%)	41 (89.13%)	
Female	13 (26.53%)	5 (10.87%)	
T stage			0.266
T1	2 (4.08%)	1 (2.17%)	
T2	14 (28.57%)	5 (10.87%)	
T3	29 (59.18%)	35 (76.09%)	
T4	1 (2.04%)	1 (2.17%)	
NA	3 (6.12%)	4 (8.70%)	
N stage			0.005**
N0	30 (61.22%)	17 (36.96%)	
N1	15 (30.61%)	13 (28.26%)	
N2	2 (4.08%)	14 (30.43%)	
N3	2 (4.08%)	2 (4.35%)	
TNM stage			0.093
I	5 (10.20%)	1 (2.17%)	
II	24 (48.98%)	15 (32.61%)	
III	14 (28.57%)	25 (54.35%)	
IV	3 (6.12%)	3 (6.52%)	
NA	3 (6.12%)	2 (4.35%)	
Tumor grade			0.084
Well	10 (20.41%)	3 (6.52%)	
Poorly	10 (20.41%)	7 (15.22%)	
Moderately	29 (59.18%)	36 (78.26%)	
IHC area% of CD38			<0.001***
<1%	10 (20.41%)	33 (71.74%)	
≥1%	39 (79.59%)	13 (28.26%)	
IHC score of INPP5E			0.083
<2	19 (38.78%)	26 (56.52%)	
≥2	30 (61.22%)	20 (43.48%)	
IHC score of POLR3G			<0.001***
<2	11 (22.45%)	37 (80.43%)	
≥2	38 (77.55%)	9 (19.57%)	
Death at FU			0.002**
No	23 (46.94%)	8 (17.39%)	
Yes	26 (53.06%)	38 (82.61%)	
OS	40.98 ± 27.01	22.52 ± 23.52	<0.001***

**p < 0.01; ***p < 0.001.

### The Clinical Predictive Ability of the Three-Gene Prognosis Signature

**Figure 5:**
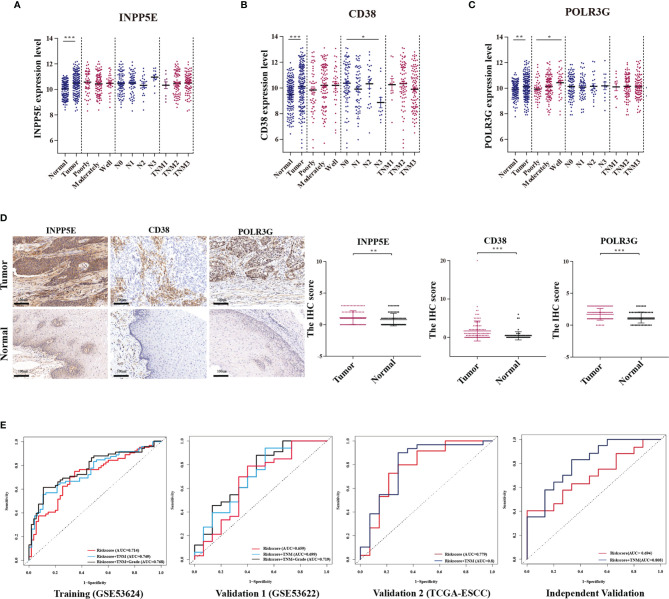
The relationship between the metabolism-related gene signature and clinicopathologic parameters. **(A‒C)** Relationships between the expression of three genes (INPP5E, CD38 and POLR3G) and the tumor characteristics, including comparison with normal tissues, tumor grade (poorly, moderately and well), N stage (N0, N1, N2 and N3) and TNM stage (TNM1, TNM2 and TNM3) were analyzed. **(D)** The differential expression between tumor and normal tissues was verified by IHC analysis. **(E)** The ROC curve of each parameter with AUC scores in the training cohort, validation 1 cohort (GSE53622), validation 2 cohort (TCGA-ESCC) and independent validation cohort. *p < 0.05; **p < 0.01; ***p < 0.001.

We analyzed the relationship between our signature and clinical factors on 179 samples from the GEO database. First, we found that the expression of CD38, INPP5E and POLR3G was significantly different between tumor and normal tissues (**Figures 5A‒C**). IHC analysis also showed differences in protein expression level (**Figure 5D**). In addition, we also found that CD38 had low expression in tumor tissue and N3 stage (**Figure 5B**). POLR3G had the lowest expression in high grade tumors (**Figure 5C**). We next found that the risk score was associated with tumor grade (**Supplementary Figure S3**). Moreover, we analyzed the sensitivity and specificity of the risk score combined with TNM stage and tumor grade through ROC curve analysis. Our results showed that the AUC value could be enhanced in the training, validation and IHC validation groups when these variables were combined (**Figure 5E** and **Supplementary Figure S4A**). Multivariate Cox analysis also showed that risk score was an independent predictive factor for OS in the IHC validation group (HR =1.874; 95% CI: 1.073-3.273; *p*=0.027; **Supplementary Figure S4B**). These results all indicated that our prognostic signature could be used as a prognostic biomarker.

In addition, we analyzed the application ability of our signature in other types of tumors from the TCGA database. Our results showed that the prognostic signature could also distinguish risk groups in head and neck squamous cell carcinoma (*p* < 0.001, **Supplementary Figure S5A**) and skin cutaneous melanoma (*p*=0.004, **Supplementary Figure S5B**). These results indicated that our signature might also be used for tumors with similar tumor characteristics but needs further exploration.

### GSEA Enrichment and Immune Infiltration Analysis of Our Signature

**Figure 6:**
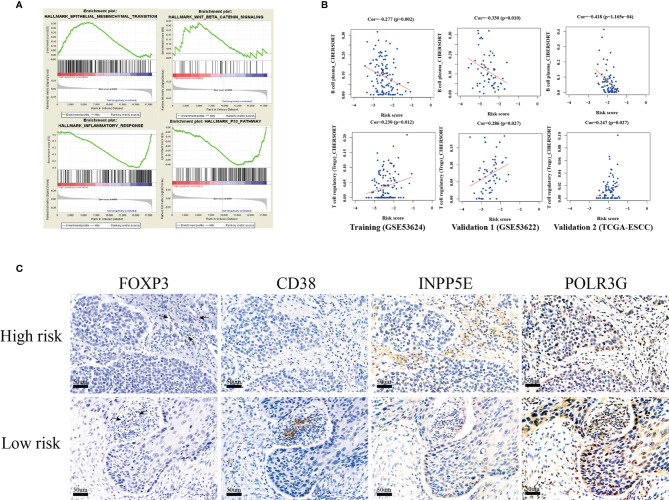
GSEA functional enrichment and immune infiltration analysis. **(A)** The four hallmark pathways were all enriched in the training, validation 1 (GSE53622) and validation 2 (TCGA-ESCC) groups by GSEA functional enrichment analysis. **(B)** The risk score was positively correlated with Treg cell infiltration, and negatively correlated with plasma cells in the training, validation 1 cohort(GSE53622) and validation 2 cohorts (TCGA-ESCC). **(C)** IHC validation of three-gene metabolic-related signatures (INPP5E,CD38 and POLR3G) and FOXP3(Treg) in 20 ESCC tissues.

The GSEA functional enrichment analysis showed that epithelial-mesenchymal transition (EMT) and Wnt-beta-catenin signaling were enriched in the high-risk group, while inflammatory response and the P53 pathway were enriched in the low-risk group; these results were consistent in the training and two validation groups (**Figure 6A**). In addition, we tried to analyze the correlation between risk score and immune infiltration and found that plasma B cell infiltration was negatively correlated with risk score and that Treg infiltration was positively correlated with risk score in the training and two validation groups (**Figure 6B**). Hence, we next analyzed the expression of FOXP3, which is a natural marker for Tregs, by IHC in 119 ESCC patient tissues belonging to the training group. Then, we selected 20 ESCC patients with higher infiltration of Tregs than others. Among these patients, we found that 15 patients belonged to our high-risk group. In addition, we found that CD38 (13/20; area percentage ≤ 1%) and POLR3G (15/20; IHC score ≤ 1) were expressed at a low level in the high-risk group, while INPP5E (12/20; IHC score > 1) was expressed at a high level (**Figure 6C**). These results all indicated that our three-gene prognosis signature involves several signaling pathways, performs well in distinguishing risk groups and is associated with tumor immune infiltration.

## Discussion

Metabolic reprogramming represents tumor-associated metabolic changes that have been regarded as hallmarks of cancer (2). A variety of metabolic changes have been found in cancer cells. A previous study showed that several tumor-associated metabolic pathways, such as glutamine metabolism, uridine metabolism, fatty acid biosynthesis, and abnormally expressed enzymes, have also been discovered in ESCC through metabolomics analysis (14). Identification of metabolic alterations is key to understanding tumor progression and exploring metabolic targeted strategies for treatment. In addition, several metabolism-related genes can also serve as diagnostic or prognostic markers.

**Figure 7:**
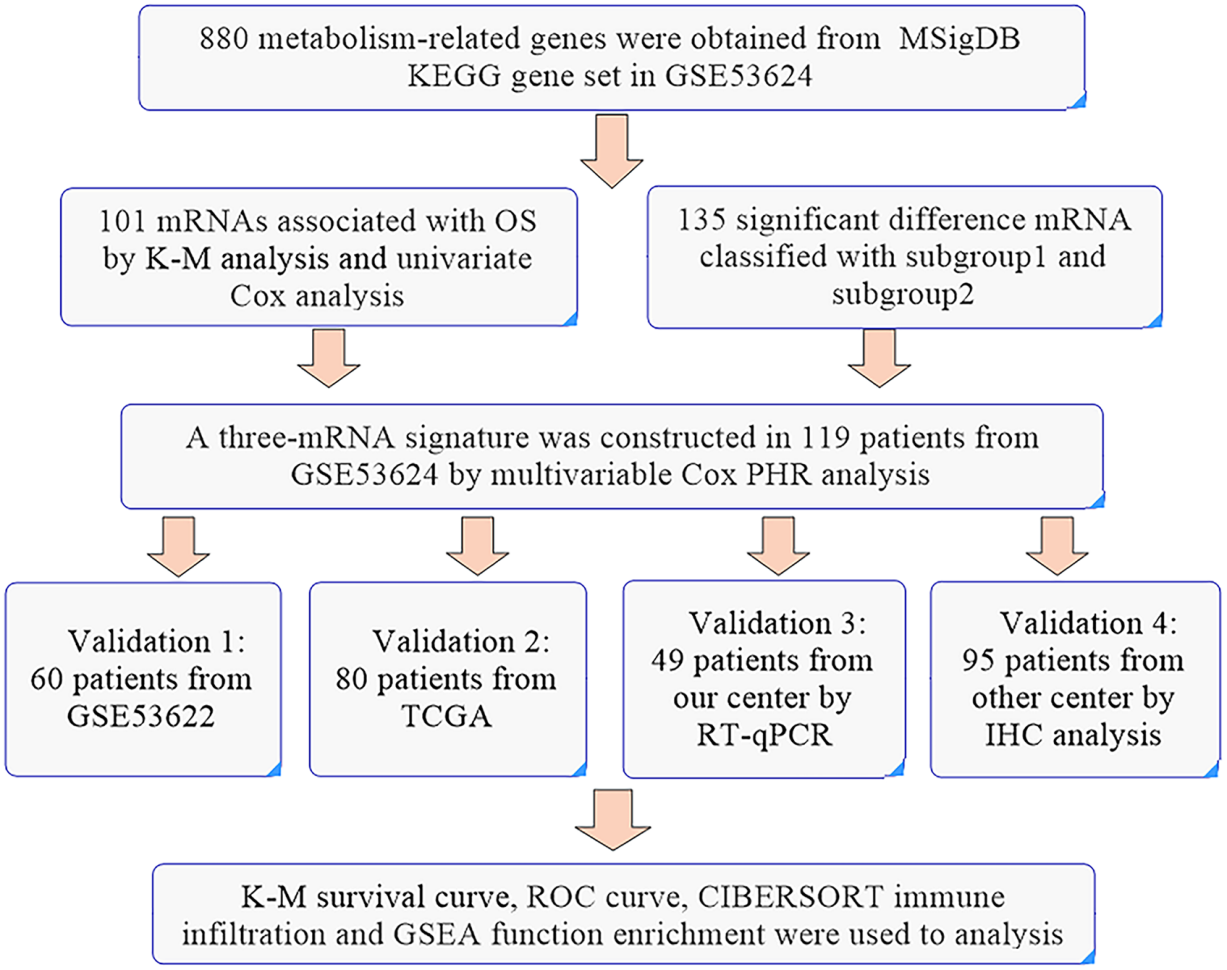
The workflow of the identification of a three-gene metabolism-related signature.

For ESCC, a multiple index assessment model was used for mortality risk prediction in cancer patients based on the expression of lncRNAs, microRNAs and mRNAs. Metabolic reprogramming is a characteristic of cancer, and a metabolic signature might be used to reflect tumor progression and patient prognosis. A previous study established a glucose-related prognostic signature for patients with glioblastoma and displayed good predictive ability (6). Glycolytic and cholesterogenic genes were used to identify distinct subgroups associated with survival and prognostic subtypes in pancreatic cancer (7). In our study, prognosis-related metabolic genes could divide ESCC patients into two subgroups. For further analysis, we found that subgroup 2 was significantly associated with poor tumor grade and OS. Hence, we established an optimal prognosis model based on prognosis-related and differential genes and the detailed workflow is shown in **Figure 7**. The three-gene prognosis signature showed a better predictive ability, which was validated by other databases. The same estimated regression coefficients and best cutoff values could also be used for the IHC validation in other center ESCC tissue arrays. Interestingly, we also found effective predictive power for other squamous cell carcinomas, such as head and neck squamous cell carcinoma and skin cutaneous melanoma. GSEA showed that several common tumor-related pathways, such as the EMT and Wnt pathways, were enriched in the high-risk group, while the inflammatory response and p53 pathways were enriched in the low-risk group. These results indicated that our prognostic signature might be specific suitable for squamous cell carcinoma and indicated similar biological characteristic changes. These genes might play an important function in tumor progression and are worth further exploration.

For the metabolism-related gene signature, CD38 is a multifunctional ectoenzyme and key modulator of nicotinamide dinucleotide (NAD+) and could also serve as a second messenger enzyme for the synthesis of cADPR. Increased CD38 activity could result in a low level of intracellular NAD+ and inhibit tumor cell growth, inducing apoptosis (15, 16). The previous studies showed that non-coding RNA expression was associated with tumor immune infiltrate (17–19). Moreover, CD38 could also play a significant role in the immunometabolism of the tumor microenvironment (15, 20). Chatterjee et al. demonstrated that metabolic reprogramming of the CD38-NAD+ axis improved T cell survival, immune cell recruitment to the tumor and T cell memory (20). INPP5E, encoding inositol polyphosphate-5-phosphatase, promotes sonic hedgehog (SHH) signaling in SHH medulloblastoma by negatively regulating a phosphoinositide 3-kinase (PI3K) signaling axis that maintains primary cilia on tumor cells (21). POLR3G is a DNA-directed RNA polymerase III subunit that is enriched in stem and cancer cells (22). POLR3G is associated with differentiation, and targeting PLOR3G might promote differentiation to reduce tumorigenicity (23). The differential expression of three genes between tumor and normal tissue was verified in the database and IHC and was also associated with OS. Three genes have direct or indirect metabolic functions and are involved in tumor progression. The prognosis signature based on three genes displays good predictive ability and serves as an independent prognostic risk factor. The clinical prognostic factors TNM stage and tumor grade were the main prognostic risk factors and could also enhance the predictive ability when combined with our signature. These results indicated that the prognostic signature has potential application value for distinguishing different ESCC subgroups and for guiding individualized treatment.

The tumor microenvironment contains several cell types, such as fibroblasts, immune cells, adipocytes and endothelial cells. Metabolic factors are generally assumed to be the reason for immunosuppression in the extracellular milieu of tumors (24). For example, tumor cells can release the immunosuppressive metabolite adenosine or tryptophan catabolite kynurenine to affect the T cell immune response (25, 26). Lactate mainly originating from tumors can drive T cells toward an immunosuppressive Treg phenotype or inhibit M2 macrophage cells (27, 28). In addition, pH and pO2 are all key players in tumor metabolism and have an effect on the tumor microenvironment and antitumor immunity (29, 30). In our study, we also found that several immune cell infiltrates were significantly associated with prognostic signature. Tregs are mainly inhibitory immune cells that have a positive correlation with our signature risk score. IHC analysis showed that the high-risk patients had a high level of infiltration of Tregs. We also found that plasma cells had a negative correlation with the risk score. CD38 is generally expressed at low levels in tissue but is particularly highly expressed in plasma cells (31). Plasma cells are characterized by coexpression of CD138 and CD38 (32). Hence, we used the expression of CD38 to analyze the infiltration of plasma cells and found that significantly low expression of CD38 was shown in the high-risk group. Altogether, these results supported that metabolism-related genes might be associated with the tumor microenvironment that regulates tumor progression.

In conclusion, our study demonstrated that metabolism-related prognostic genes could stratify patients into two subgroups and were associated with tumor grade and OS. The three-gene prognostic signature from metabolism-related genes displays a good ability to predict OS and the infiltration of immunosuppressive Tregs and plasma cells.

## Data Availability Statement

Publicly available datasets were analyzed in this study. This data can be found here: The public datasets GSE53622,GSE53624 and TCGA-ESCC and clinical information for this study can be found in the GEO and TCGA database.

## Ethics Statement

The studies involving human participants were reviewed and approved by Medical ethics committee of the Cancer institute and Hospital, Chinese Academy of Medical Sciences. The patients/participants provided their written informed consent to participate in this study. Written informed consent was obtained from the individual(s) for the publication of any potentially identifiable images or data included in this article.

## Author Contributions

YL, CL, and JH design this study. YL, LW, LF, HL, HT, YZ, and TF collect and analyze the public database. All authors read and approved the final manuscript.

## Funding

This work was supported by the National Natural Science Foundation of China (81972196), the National Key R&D Program of China (2020AAA0109505) and the R&D Program of Beijing Municipal Education commission (KJZD20191002302).

## Conflict of Interest

The authors declare that the research was conducted in the absence of any commercial or financial relationships that could be construed as a potential conflict of interest.

## Publisher’s Note

All claims expressed in this article are solely those of the authors and do not necessarily represent those of their affiliated organizations, or those of the publisher, the editors and the reviewers. Any product that may be evaluated in this article, or claim that may be made by its manufacturer, is not guaranteed or endorsed by the publisher.
