# Supplementary material for: A Multi-Center Validated Subtyping Model of Esophageal Cancer Based on Three Metabolism-Related Genes
Source: Front Oncol. 2021 Oct 25;11:772145. doi: 10.3389/fonc.2021.772145 (PMC8573269; doi:10.3389/fonc.2021.772145)
Supplement: Supplementary file 1 [file DataSheet_1.docx]

Supplementary Material

## Supplementary Figures


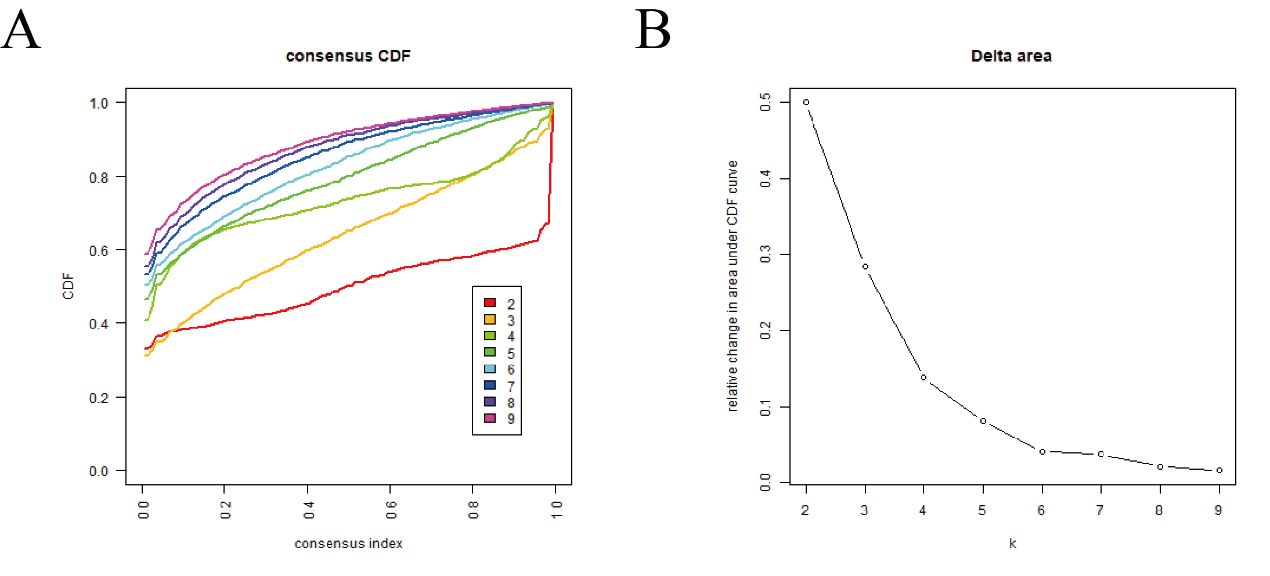


**Supplementary Figure S1.** **The consensus clustering analysis results.** The consensus cumulative distribution function (CDF) and delta area from k = 2 to k = 10.

**
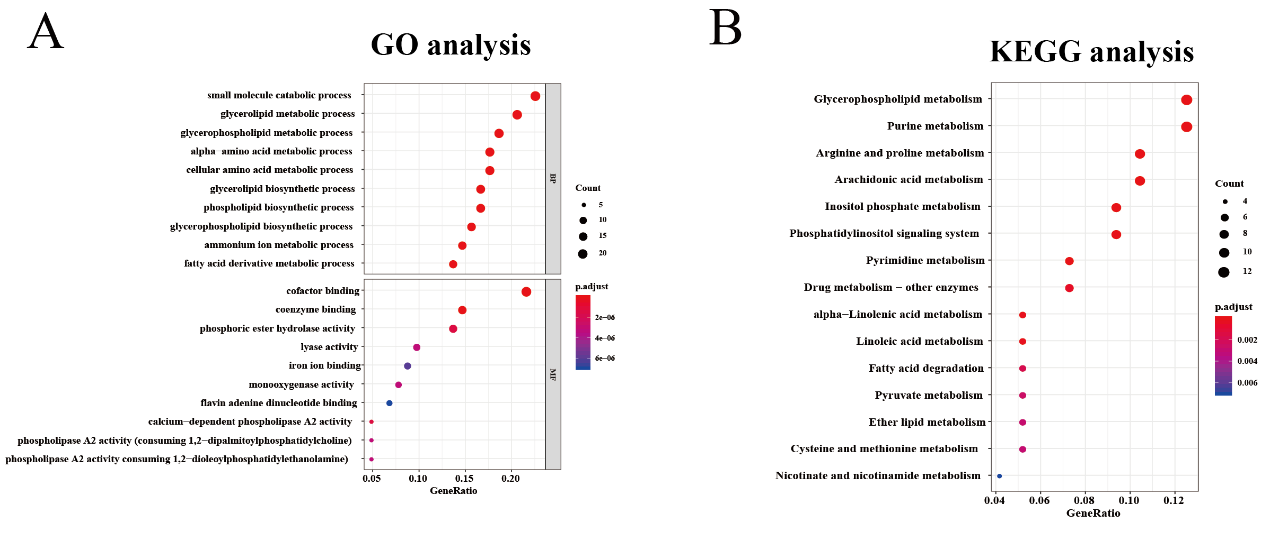
**

**Supplementary Figure S2.** **The function enrichment analysis of 101 prognosis-related metabolic gene by DAVID.** (A)The GO function analysis of 101 prognosis-related metabolic gene. (B) The KEGG signaling pathway analysis of 101 prognosis-related metabolic gene by DAVID.


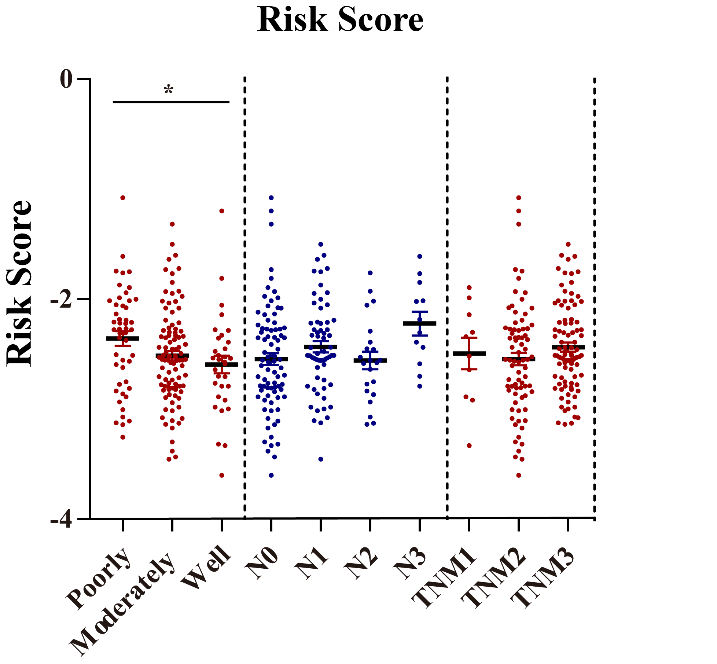


**Supplementary Figure S3.** **The relationship between risk score and clinical factors in training cohort.**


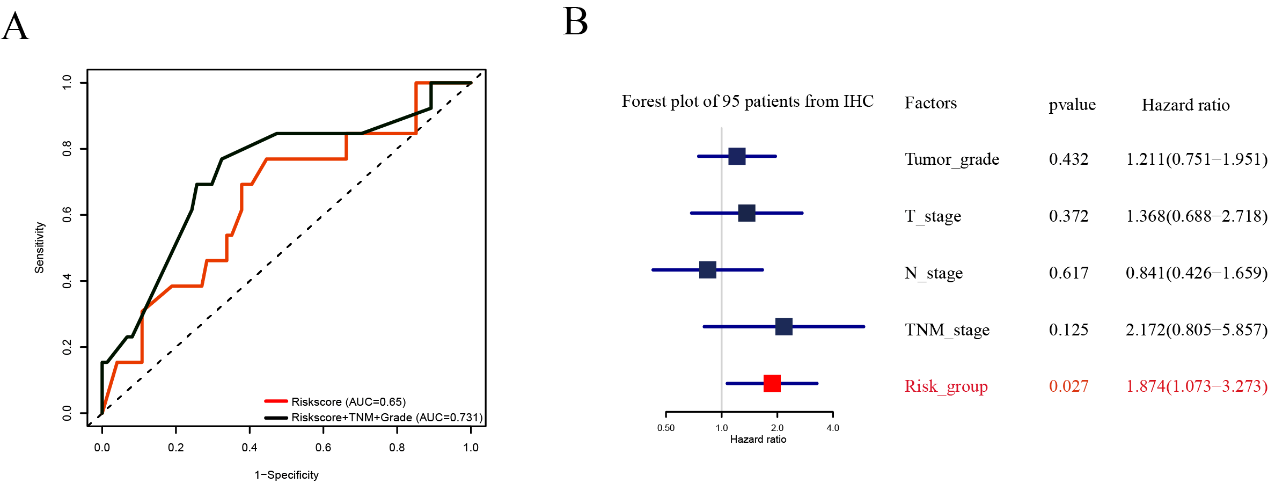


**Supplementary Figure S4. The validation of prognosis signature in IHC validation cohort.** (A) The ROC curve with AUC scores in IHC validation cohort. (B) The multivariate Cox regression analysis in IHC validation cohort.


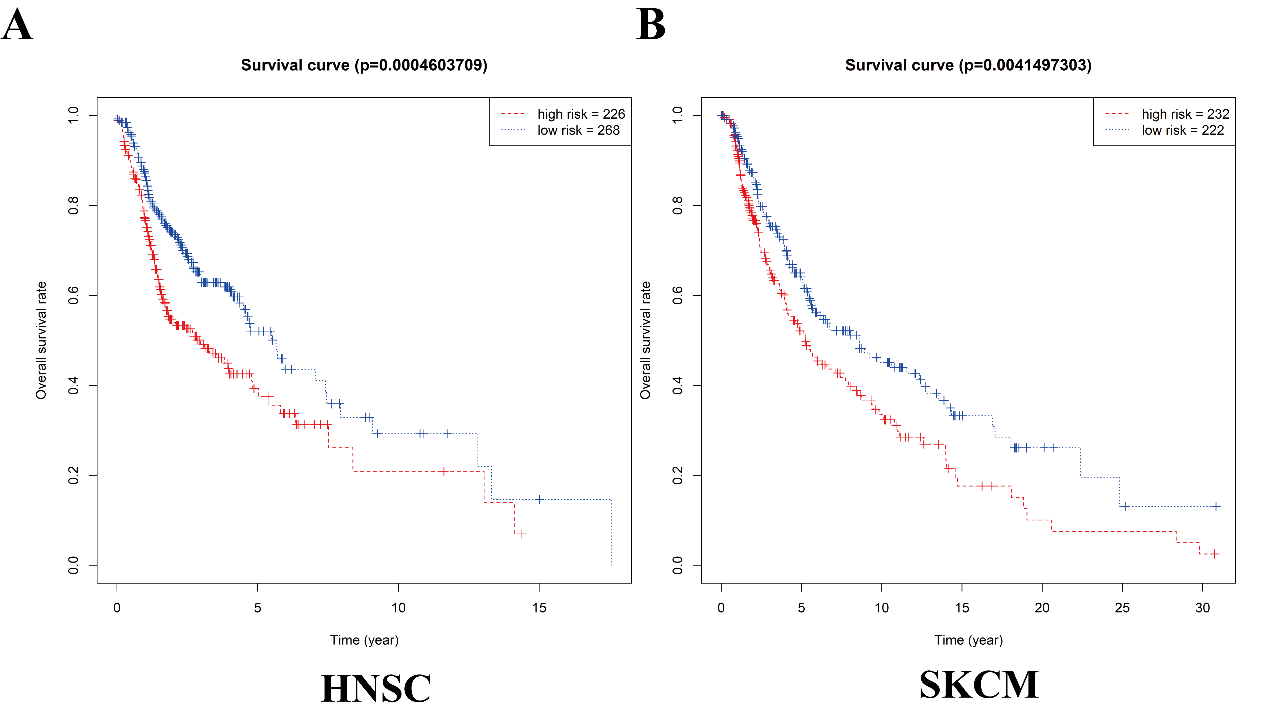


**Supplementary Figure S5. The verification results of three gene prognosis signature in head and neck squamous cell carcinoma and skin cutaneous melanoma from TCGA.** (A)The Kaplan-Meier survival analysis of overall survival in head and neck squamous cell carcinoma. (B) The Kaplan-Meier survival analysis of overall survival in skin cutaneous melanoma.

**Supplementary Table S1 The metabolism-related gene list**

| **Gene Name** | **Species** | **Description** |
| --- | --- | --- |
| AACS | Homo sapiens | acetoacetyl-CoA synthetase(AACS) |
| AADAT | Homo sapiens | aminoadipate aminotransferase(AADAT) |
| AANAT | Homo sapiens | aralkylamine N-acetyltransferase(AANAT) |
| ABAT | Homo sapiens | 4-aminobutyrate aminotransferase(ABAT) |
| ACAA2 | Homo sapiens | acetyl-CoA acyltransferase 2(ACAA2) |
| ACACA | Homo sapiens | acetyl-CoA carboxylase alpha(ACACA) |
| ACACB | Homo sapiens | acetyl-CoA carboxylase beta(ACACB) |
| ACADL | Homo sapiens | acyl-CoA dehydrogenase, long chain(ACADL) |
| ACADM | Homo sapiens | acyl-CoA dehydrogenase, C-4 to C-12 straight chain(ACADM) |
| ACADS | Homo sapiens | acyl-CoA dehydrogenase, C-2 to C-3 short chain(ACADS) |
| ACADSB | Homo sapiens | acyl-CoA dehydrogenase, short/branched chain(ACADSB) |
| ACADVL | Homo sapiens | acyl-CoA dehydrogenase, very long chain(ACADVL) |
| ACAT1 | Homo sapiens | acetyl-CoA acetyltransferase 1(ACAT1) |
| ACAT2 | Homo sapiens | acetyl-CoA acetyltransferase 2(ACAT2) |
| ACER1 | Homo sapiens | alkaline ceramidase 1(ACER1) |
| ACER2 | Homo sapiens | alkaline ceramidase 2(ACER2) |
| ACER3 | Homo sapiens | alkaline ceramidase 3(ACER3) |
| ACHE | Homo sapiens | acetylcholinesterase (Cartwright blood group)(ACHE) |
| ACMSD | Homo sapiens | aminocarboxymuconate semialdehyde decarboxylase(ACMSD) |
| ACO1 | Homo sapiens | aconitase 1(ACO1) |
| ACO2 | Homo sapiens | aconitase 2(ACO2) |
| ACOT12 | Homo sapiens | acyl-CoA thioesterase 12(ACOT12) |
| ACOX1 | Homo sapiens | acyl-CoA oxidase 1(ACOX1) |
| ACOX3 | Homo sapiens | acyl-CoA oxidase 3, pristanoyl(ACOX3) |
| ACP1 | Homo sapiens | acid phosphatase 1, soluble(ACP1) |
| ACP2 | Homo sapiens | acid phosphatase 2, lysosomal(ACP2) |
| ACP4 | Homo sapiens | acid phosphatase 4(ACP4) |
| ACP5 | Homo sapiens | acid phosphatase 5, tartrate resistant(ACP5) |
| ACP6 | Homo sapiens | acid phosphatase 6, lysophosphatidic(ACP6) |
| ACPP | Homo sapiens | acid phosphatase, prostate(ACPP) |
| ACSL1 | Homo sapiens | acyl-CoA synthetase long-chain family member 1(ACSL1) |
| ACSL3 | Homo sapiens | acyl-CoA synthetase long-chain family member 3(ACSL3) |
| ACSL4 | Homo sapiens | acyl-CoA synthetase long-chain family member 4(ACSL4) |
| ACSL5 | Homo sapiens | acyl-CoA synthetase long-chain family member 5(ACSL5) |
| ACSL6 | Homo sapiens | acyl-CoA synthetase long-chain family member 6(ACSL6) |
| ACSM1 | Homo sapiens | acyl-CoA synthetase medium-chain family member 1(ACSM1) |
| ACSM2A | Homo sapiens | acyl-CoA synthetase medium-chain family member 2A(ACSM2A) |
| ACSM3 | Homo sapiens | acyl-CoA synthetase medium-chain family member 3(ACSM3) |
| ACSM4 | Homo sapiens | acyl-CoA synthetase medium-chain family member 4(ACSM4) |
| ACSM5 | Homo sapiens | acyl-CoA synthetase medium-chain family member 5(ACSM5) |
| ACSS1 | Homo sapiens | acyl-CoA synthetase short-chain family member 1(ACSS1) |
| ACSS2 | Homo sapiens | acyl-CoA synthetase short-chain family member 2(ACSS2) |
| ACSS3 | Homo sapiens | acyl-CoA synthetase short-chain family member 3(ACSS3) |
| ACY1 | Homo sapiens | aminoacylase 1(ACY1) |
| ACY3 | Homo sapiens | aminoacylase 3(ACY3) |
| ACYP1 | Homo sapiens | acylphosphatase 1(ACYP1) |
| ACYP2 | Homo sapiens | acylphosphatase 2(ACYP2) |
| ADA | Homo sapiens | adenosine deaminase(ADA) |
| ADCY10 | Homo sapiens | adenylate cyclase 10, soluble(ADCY10) |
| ADCY2 | Homo sapiens | adenylate cyclase 2(ADCY2) |
| ADCY4 | Homo sapiens | adenylate cyclase 4(ADCY4) |
| ADCY5 | Homo sapiens | adenylate cyclase 5(ADCY5) |
| ADCY6 | Homo sapiens | adenylate cyclase 6(ADCY6) |
| ADCY8 | Homo sapiens | adenylate cyclase 8(ADCY8) |
| ADCY9 | Homo sapiens | adenylate cyclase 9(ADCY9) |
| ADH1B | Homo sapiens | alcohol dehydrogenase 1B (class I), beta polypeptide(ADH1B) |
| ADH1C | Homo sapiens | alcohol dehydrogenase 1C (class I), gamma polypeptide(ADH1C) |
| ADH5 | Homo sapiens | alcohol dehydrogenase 5 (class III), chi polypeptide(ADH5) |
| ADH6 | Homo sapiens | alcohol dehydrogenase 6 (class V)(ADH6) |
| ADH7 | Homo sapiens | alcohol dehydrogenase 7 (class IV), mu or sigma polypeptide(ADH7) |
| ADI1 | Homo sapiens | acireductone dioxygenase 1(ADI1) |
| ADK | Homo sapiens | adenosine kinase(ADK) |
| ADO | Homo sapiens | 2-aminoethanethiol dioxygenase(ADO) |
| ADPRM | Homo sapiens | ADP-ribose/CDP-alcohol diphosphatase, manganese dependent(ADPRM) |
| ADSL | Homo sapiens | adenylosuccinate lyase(ADSL) |
| ADSS | Homo sapiens | adenylosuccinate synthase(ADSS) |
| ADSSL1 | Homo sapiens | adenylosuccinate synthase like 1(ADSSL1) |
| AFMID | Homo sapiens | arylformamidase(AFMID) |
| AGK | Homo sapiens | acylglycerol kinase(AGK) |
| AGL | Homo sapiens | amylo-alpha-1, 6-glucosidase, 4-alpha-glucanotransferase(AGL) |
| AGMAT | Homo sapiens | agmatinase(AGMAT) |
| AGPAT1 | Homo sapiens | 1-acylglycerol-3-phosphate O-acyltransferase 1(AGPAT1) |
| AGPAT2 | Homo sapiens | 1-acylglycerol-3-phosphate O-acyltransferase 2(AGPAT2) |
| AGPAT3 | Homo sapiens | 1-acylglycerol-3-phosphate O-acyltransferase 3(AGPAT3) |
| AGPAT4 | Homo sapiens | 1-acylglycerol-3-phosphate O-acyltransferase 4(AGPAT4) |
| AGPS | Homo sapiens | alkylglycerone phosphate synthase(AGPS) |
| AGXT | Homo sapiens | alanine-glyoxylate aminotransferase(AGXT) |
| AGXT2 | Homo sapiens | alanine--glyoxylate aminotransferase 2(AGXT2) |
| AHCY | Homo sapiens | adenosylhomocysteinase(AHCY) |
| AHCYL1 | Homo sapiens | adenosylhomocysteinase like 1(AHCYL1) |
| AHCYL2 | Homo sapiens | adenosylhomocysteinase like 2(AHCYL2) |
| AK1 | Homo sapiens | adenylate kinase 1(AK1) |
| AK2 | Homo sapiens | adenylate kinase 2(AK2) |
| AK3 | Homo sapiens | adenylate kinase 3(AK3) |
| AK4 | Homo sapiens | adenylate kinase 4(AK4) |
| AK5 | Homo sapiens | adenylate kinase 5(AK5) |
| AK7 | Homo sapiens | adenylate kinase 7(AK7) |
| AKR1A1 | Homo sapiens | aldo-keto reductase family 1 member A1(AKR1A1) |
| AKR1B1 | Homo sapiens | aldo-keto reductase family 1 member B(AKR1B1) |
| AKR1B10 | Homo sapiens | aldo-keto reductase family 1 member B10(AKR1B10) |
| AKR1C1 | Homo sapiens | aldo-keto reductase family 1 member C1(AKR1C1) |
| AKR1C3 | Homo sapiens | aldo-keto reductase family 1 member C3(AKR1C3) |
| AKR1C4 | Homo sapiens | aldo-keto reductase family 1 member C4(AKR1C4) |
| ALAD | Homo sapiens | aminolevulinate dehydratase(ALAD) |
| ALAS1 | Homo sapiens | 5'-aminolevulinate synthase 1(ALAS1) |
| ALAS2 | Homo sapiens | 5'-aminolevulinate synthase 2(ALAS2) |
| ALDH18A1 | Homo sapiens | aldehyde dehydrogenase 18 family member A1(ALDH18A1) |
| ALDH1A1 | Homo sapiens | aldehyde dehydrogenase 1 family member A1(ALDH1A1) |
| ALDH1A2 | Homo sapiens | aldehyde dehydrogenase 1 family member A2(ALDH1A2) |
| ALDH1A3 | Homo sapiens | aldehyde dehydrogenase 1 family member A3(ALDH1A3) |
| ALDH1B1 | Homo sapiens | aldehyde dehydrogenase 1 family member B1(ALDH1B1) |
| ALDH2 | Homo sapiens | aldehyde dehydrogenase 2 family (mitochondrial)(ALDH2) |
| ALDH3A1 | Homo sapiens | aldehyde dehydrogenase 3 family member A1(ALDH3A1) |
| ALDH3A2 | Homo sapiens | aldehyde dehydrogenase 3 family member A2(ALDH3A2) |
| ALDH3B1 | Homo sapiens | aldehyde dehydrogenase 3 family member B1(ALDH3B1) |
| ALDH3B2 | Homo sapiens | aldehyde dehydrogenase 3 family member B2(ALDH3B2) |
| ALDH4A1 | Homo sapiens | aldehyde dehydrogenase 4 family member A1(ALDH4A1) |
| ALDH5A1 | Homo sapiens | aldehyde dehydrogenase 5 family member A1(ALDH5A1) |
| ALDH6A1 | Homo sapiens | aldehyde dehydrogenase 6 family member A1(ALDH6A1) |
| ALDH7A1 | Homo sapiens | aldehyde dehydrogenase 7 family member A1(ALDH7A1) |
| ALDH9A1 | Homo sapiens | aldehyde dehydrogenase 9 family member A1(ALDH9A1) |
| ALDOA | Homo sapiens | aldolase, fructose-bisphosphate A(ALDOA) |
| ALDOB | Homo sapiens | aldolase, fructose-bisphosphate B(ALDOB) |
| ALDOC | Homo sapiens | aldolase, fructose-bisphosphate C(ALDOC) |
| ALLC | Homo sapiens | allantoicase(ALLC) |
| ALOX12 | Homo sapiens | arachidonate 12-lipoxygenase, 12S type(ALOX12) |
| ALOX12B | Homo sapiens | arachidonate 12-lipoxygenase, 12R type(ALOX12B) |
| ALOX15 | Homo sapiens | arachidonate 15-lipoxygenase(ALOX15) |
| ALOX15B | Homo sapiens | arachidonate 15-lipoxygenase, type B(ALOX15B) |
| ALOX5 | Homo sapiens | arachidonate 5-lipoxygenase(ALOX5) |
| AMD1 | Homo sapiens | adenosylmethionine decarboxylase 1(AMD1) |
| AMDHD1 | Homo sapiens | amidohydrolase domain containing 1(AMDHD1) |
| AMDHD2 | Homo sapiens | amidohydrolase domain containing 2(AMDHD2) |
| AMPD1 | Homo sapiens | adenosine monophosphate deaminase 1(AMPD1) |
| AMPD2 | Homo sapiens | adenosine monophosphate deaminase 2(AMPD2) |
| AMPD3 | Homo sapiens | adenosine monophosphate deaminase 3(AMPD3) |
| AMT | Homo sapiens | aminomethyltransferase(AMT) |
| AMY1B | Homo sapiens | amylase, alpha 1B (salivary)(AMY1B) |
| AMY2B | Homo sapiens | amylase, alpha 2B (pancreatic)(AMY2B) |
| ANPEP | Homo sapiens | alanyl aminopeptidase, membrane(ANPEP) |
| AOC1 | Homo sapiens | amine oxidase, copper containing 1(AOC1) |
| AOC2 | Homo sapiens | amine oxidase, copper containing 2(AOC2) |
| AOC3 | Homo sapiens | amine oxidase, copper containing 3(AOC3) |
| AOX1 | Homo sapiens | aldehyde oxidase 1(AOX1) |
| APIP | Homo sapiens | APAF1 interacting protein(APIP) |
| APRT | Homo sapiens | adenine phosphoribosyltransferase(APRT) |
| ARG1 | Homo sapiens | arginase 1(ARG1) |
| ARSA | Homo sapiens | arylsulfatase A(ARSA) |
| ASAH1 | Homo sapiens | N-acylsphingosine amidohydrolase 1(ASAH1) |
| ASL | Homo sapiens | argininosuccinate lyase(ASL) |
| ASMT | Homo sapiens | acetylserotonin O-methyltransferase(ASMT) |
| ASPA | Homo sapiens | aspartoacylase(ASPA) |
| ASS1 | Homo sapiens | argininosuccinate synthase 1(ASS1) |
| ATIC | Homo sapiens | 5-aminoimidazole-4-carboxamide ribonucleotide formyltransferase/IMP cyclohydrolase(ATIC) |
| AWAT2 | Homo sapiens | acyl-CoA wax alcohol acyltransferase 2(AWAT2) |
| AZIN2 | Homo sapiens | antizyme inhibitor 2(AZIN2) |
| B4GALT1 | Homo sapiens | beta-1,4-galactosyltransferase 1(B4GALT1) |
| B4GALT2 | Homo sapiens | beta-1,4-galactosyltransferase 2(B4GALT2) |
| B4GALT6 | Homo sapiens | beta-1,4-galactosyltransferase 6(B4GALT6) |
| BAAT | Homo sapiens | bile acid-CoA:amino acid N-acyltransferase(BAAT) |
| BCO1 | Homo sapiens | beta-carotene oxygenase 1(BCO1) |
| BDH1 | Homo sapiens | 3-hydroxybutyrate dehydrogenase, type 1(BDH1) |
| BHMT | Homo sapiens | betaine--homocysteine S-methyltransferase(BHMT) |
| BLVRA | Homo sapiens | biliverdin reductase A(BLVRA) |
| BLVRB | Homo sapiens | biliverdin reductase B(BLVRB) |
| BPNT1 | Homo sapiens | 3'(2'), 5'-bisphosphate nucleotidase 1(BPNT1) |
| BST1 | Homo sapiens | bone marrow stromal cell antigen 1(BST1) |
| BUD23 | Homo sapiens | BUD23 rRNA methyltransferase and ribosome maturation factor(BUD23) |
| CA1 | Homo sapiens | carbonic anhydrase 1(CA1) |
| CA12 | Homo sapiens | carbonic anhydrase 12(CA12) |
| CA13 | Homo sapiens | carbonic anhydrase 13(CA13) |
| CA14 | Homo sapiens | carbonic anhydrase 14(CA14) |
| CA2 | Homo sapiens | carbonic anhydrase 2(CA2) |
| CA3 | Homo sapiens | carbonic anhydrase 3(CA3) |
| CA4 | Homo sapiens | carbonic anhydrase 4(CA4) |
| CA5B | Homo sapiens | carbonic anhydrase 5B(CA5B) |
| CA6 | Homo sapiens | carbonic anhydrase 6(CA6) |
| CA7 | Homo sapiens | carbonic anhydrase 7(CA7) |
| CA8 | Homo sapiens | carbonic anhydrase 8(CA8) |
| CA9 | Homo sapiens | carbonic anhydrase 9(CA9) |
| CAD | Homo sapiens | carbamoyl-phosphate synthetase 2, aspartate transcarbamylase, and dihydroorotase(CAD) |
| CANT1 | Homo sapiens | calcium activated nucleotidase 1(CANT1) |
| CAT | Homo sapiens | catalase(CAT) |
| CBR1 | Homo sapiens | carbonyl reductase 1(CBR1) |
| CBR3 | Homo sapiens | carbonyl reductase 3(CBR3) |
| CD38 | Homo sapiens | CD38 molecule(CD38) |
| CDA | Homo sapiens | cytidine deaminase(CDA) |
| CDIPT | Homo sapiens | CDP-diacylglycerol--inositol 3-phosphatidyltransferase(CDIPT) |
| CDO1 | Homo sapiens | cysteine dioxygenase type 1(CDO1) |
| CDS1 | Homo sapiens | CDP-diacylglycerol synthase 1(CDS1) |
| CDS2 | Homo sapiens | CDP-diacylglycerol synthase 2(CDS2) |
| CEL | Homo sapiens | carboxyl ester lipase(CEL) |
| CERK | Homo sapiens | ceramide kinase(CERK) |
| CES1 | Homo sapiens | carboxylesterase 1(CES1) |
| CES5A | Homo sapiens | carboxylesterase 5A(CES5A) |
| CHAT | Homo sapiens | choline O-acetyltransferase(CHAT) |
| CHDH | Homo sapiens | choline dehydrogenase(CHDH) |
| CHIA | Homo sapiens | chitinase, acidic(CHIA) |
| CHIT1 | Homo sapiens | chitinase 1(CHIT1) |
| CHPT1 | Homo sapiens | choline phosphotransferase 1(CHPT1) |
| CHST11 | Homo sapiens | carbohydrate sulfotransferase 11(CHST11) |
| CHST12 | Homo sapiens | carbohydrate sulfotransferase 12(CHST12) |
| CHST13 | Homo sapiens | carbohydrate sulfotransferase 13(CHST13) |
| CKB | Homo sapiens | creatine kinase B(CKB) |
| CKM | Homo sapiens | creatine kinase, M-type(CKM) |
| CKMT1A | Homo sapiens | creatine kinase, mitochondrial 1A(CKMT1A) |
| CKMT1B | Homo sapiens | creatine kinase, mitochondrial 1B(CKMT1B) |
| CKMT2 | Homo sapiens | creatine kinase, mitochondrial 2(CKMT2) |
| CMAS | Homo sapiens | cytidine monophosphate N-acetylneuraminic acid synthetase(CMAS) |
| CMPK1 | Homo sapiens | cytidine/uridine monophosphate kinase 1(CMPK1) |
| CMPK2 | Homo sapiens | cytidine/uridine monophosphate kinase 2(CMPK2) |
| CNDP1 | Homo sapiens | carnosine dipeptidase 1(CNDP1) |
| COMT | Homo sapiens | catechol-O-methyltransferase(COMT) |
| COX10 | Homo sapiens | COX10, heme A:farnesyltransferase cytochrome c oxidase assembly factor(COX10) |
| COX15 | Homo sapiens | COX15, cytochrome c oxidase assembly homolog(COX15) |
| CP | Homo sapiens | ceruloplasmin(CP) |
| CPOX | Homo sapiens | coproporphyrinogen oxidase(CPOX) |
| CPS1 | Homo sapiens | carbamoyl-phosphate synthase 1(CPS1) |
| CPT1A | Homo sapiens | carnitine palmitoyltransferase 1A(CPT1A) |
| CPT1B | Homo sapiens | carnitine palmitoyltransferase 1B(CPT1B) |
| CPT1C | Homo sapiens | carnitine palmitoyltransferase 1C(CPT1C) |
| CPT2 | Homo sapiens | carnitine palmitoyltransferase 2(CPT2) |
| CRLS1 | Homo sapiens | cardiolipin synthase 1(CRLS1) |
| CS | Homo sapiens | citrate synthase(CS) |
| CSAD | Homo sapiens | cysteine sulfinic acid decarboxylase(CSAD) |
| CTH | Homo sapiens | cystathionine gamma-lyase(CTH) |
| CTPS1 | Homo sapiens | CTP synthase 1(CTPS1) |
| CTPS2 | Homo sapiens | CTP synthase 2(CTPS2) |
| CYB5R1 | Homo sapiens | cytochrome b5 reductase 1(CYB5R1) |
| CYB5R3 | Homo sapiens | cytochrome b5 reductase 3(CYB5R3) |
| CYP1A1 | Homo sapiens | cytochrome P450 family 1 subfamily A member 1(CYP1A1) |
| CYP1B1 | Homo sapiens | cytochrome P450 family 1 subfamily B member 1(CYP1B1) |
| CYP26A1 | Homo sapiens | cytochrome P450 family 26 subfamily A member 1(CYP26A1) |
| CYP26B1 | Homo sapiens | cytochrome P450 family 26 subfamily B member 1(CYP26B1) |
| CYP26C1 | Homo sapiens | cytochrome P450 family 26 subfamily C member 1(CYP26C1) |
| CYP2A13 | Homo sapiens | cytochrome P450 family 2 subfamily A member 13(CYP2A13) |
| CYP2A6 | Homo sapiens | cytochrome P450 family 2 subfamily A member 6(CYP2A6) |
| CYP2A7 | Homo sapiens | cytochrome P450 family 2 subfamily A member 7(CYP2A7) |
| CYP2B6 | Homo sapiens | cytochrome P450 family 2 subfamily B member 6(CYP2B6) |
| CYP2C18 | Homo sapiens | cytochrome P450 family 2 subfamily C member 18(CYP2C18) |
| CYP2C19 | Homo sapiens | cytochrome P450 family 2 subfamily C member 19(CYP2C19) |
| CYP2C8 | Homo sapiens | cytochrome P450 family 2 subfamily C member 8(CYP2C8) |
| CYP2C9 | Homo sapiens | cytochrome P450 family 2 subfamily C member 9(CYP2C9) |
| CYP2E1 | Homo sapiens | cytochrome P450 family 2 subfamily E member 1(CYP2E1) |
| CYP2J2 | Homo sapiens | cytochrome P450 family 2 subfamily J member 2(CYP2J2) |
| CYP2S1 | Homo sapiens | cytochrome P450 family 2 subfamily S member 1(CYP2S1) |
| CYP2U1 | Homo sapiens | cytochrome P450 family 2 subfamily U member 1(CYP2U1) |
| CYP3A4 | Homo sapiens | cytochrome P450 family 3 subfamily A member 4(CYP3A4) |
| CYP3A5 | Homo sapiens | cytochrome P450 family 3 subfamily A member 5(CYP3A5) |
| CYP3A7 | Homo sapiens | cytochrome P450 family 3 subfamily A member 7(CYP3A7) |
| CYP4A11 | Homo sapiens | cytochrome P450 family 4 subfamily A member 11(CYP4A11) |
| CYP4A22 | Homo sapiens | cytochrome P450 family 4 subfamily A member 22(CYP4A22) |
| CYP4F2 | Homo sapiens | cytochrome P450 family 4 subfamily F member 2(CYP4F2) |
| CYP4F3 | Homo sapiens | cytochrome P450 family 4 subfamily F member 3(CYP4F3) |
| DAO | Homo sapiens | D-amino acid oxidase(DAO) |
| DBH | Homo sapiens | dopamine beta-hydroxylase(DBH) |
| DCK | Homo sapiens | deoxycytidine kinase(DCK) |
| DCT | Homo sapiens | dopachrome tautomerase(DCT) |
| DCTD | Homo sapiens | dCMP deaminase(DCTD) |
| DDC | Homo sapiens | dopa decarboxylase(DDC) |
| DDO | Homo sapiens | D-aspartate oxidase(DDO) |
| DEGS1 | Homo sapiens | delta 4-desaturase, sphingolipid 1(DEGS1) |
| DEGS2 | Homo sapiens | delta 4-desaturase, sphingolipid 2(DEGS2) |
| DGAT1 | Homo sapiens | diacylglycerol O-acyltransferase 1(DGAT1) |
| DGAT2 | Homo sapiens | diacylglycerol O-acyltransferase 2(DGAT2) |
| DGKA | Homo sapiens | diacylglycerol kinase alpha(DGKA) |
| DGKB | Homo sapiens | diacylglycerol kinase beta(DGKB) |
| DGKD | Homo sapiens | diacylglycerol kinase delta(DGKD) |
| DGKE | Homo sapiens | diacylglycerol kinase epsilon(DGKE) |
| DGKG | Homo sapiens | diacylglycerol kinase gamma(DGKG) |
| DGKH | Homo sapiens | diacylglycerol kinase eta(DGKH) |
| DGKI | Homo sapiens | diacylglycerol kinase iota(DGKI) |
| DGKQ | Homo sapiens | diacylglycerol kinase theta(DGKQ) |
| DGKZ | Homo sapiens | diacylglycerol kinase zeta(DGKZ) |
| DGUOK | Homo sapiens | deoxyguanosine kinase(DGUOK) |
| DHDH | Homo sapiens | dihydrodiol dehydrogenase(DHDH) |
| DHODH | Homo sapiens | dihydroorotate dehydrogenase (quinone)(DHODH) |
| DHRS3 | Homo sapiens | dehydrogenase/reductase 3(DHRS3) |
| DHRS4 | Homo sapiens | dehydrogenase/reductase 4(DHRS4) |
| DHRS4L2 | Homo sapiens | dehydrogenase/reductase 4 like 2(DHRS4L2) |
| DHRS9 | Homo sapiens | dehydrogenase/reductase 9(DHRS9) |
| DLAT | Homo sapiens | dihydrolipoamide S-acetyltransferase(DLAT) |
| DLD | Homo sapiens | dihydrolipoamide dehydrogenase(DLD) |
| DMGDH | Homo sapiens | dimethylglycine dehydrogenase(DMGDH) |
| DNMT1 | Homo sapiens | DNA methyltransferase 1(DNMT1) |
| DNMT3A | Homo sapiens | DNA methyltransferase 3 alpha(DNMT3A) |
| DNMT3B | Homo sapiens | DNA methyltransferase 3 beta(DNMT3B) |
| DNMT3L | Homo sapiens | DNA methyltransferase 3 like(DNMT3L) |
| DPYD | Homo sapiens | dihydropyrimidine dehydrogenase(DPYD) |
| DPYS | Homo sapiens | dihydropyrimidinase(DPYS) |
| DTYMK | Homo sapiens | deoxythymidylate kinase(DTYMK) |
| DUT | Homo sapiens | deoxyuridine triphosphatase(DUT) |
| EARS2 | Homo sapiens | glutamyl-tRNA synthetase 2, mitochondrial(EARS2) |
| ECHS1 | Homo sapiens | enoyl-CoA hydratase, short chain 1(ECHS1) |
| ECI1 | Homo sapiens | enoyl-CoA delta isomerase 1(ECI1) |
| ECI2 | Homo sapiens | enoyl-CoA delta isomerase 2(ECI2) |
| EHHADH | Homo sapiens | enoyl-CoA hydratase and 3-hydroxyacyl CoA dehydrogenase(EHHADH) |
| ENOPH1 | Homo sapiens | enolase-phosphatase 1(ENOPH1) |
| ENPP1 | Homo sapiens | ectonucleotide pyrophosphatase/phosphodiesterase 1(ENPP1) |
| ENPP2 | Homo sapiens | ectonucleotide pyrophosphatase/phosphodiesterase 2(ENPP2) |
| ENPP3 | Homo sapiens | ectonucleotide pyrophosphatase/phosphodiesterase 3(ENPP3) |
| ENPP6 | Homo sapiens | ectonucleotide pyrophosphatase/phosphodiesterase 6(ENPP6) |
| ENPP7 | Homo sapiens | ectonucleotide pyrophosphatase/phosphodiesterase 7(ENPP7) |
| ENTPD1 | Homo sapiens | ectonucleoside triphosphate diphosphohydrolase 1(ENTPD1) |
| ENTPD2 | Homo sapiens | ectonucleoside triphosphate diphosphohydrolase 2(ENTPD2) |
| ENTPD3 | Homo sapiens | ectonucleoside triphosphate diphosphohydrolase 3(ENTPD3) |
| ENTPD4 | Homo sapiens | ectonucleoside triphosphate diphosphohydrolase 4(ENTPD4) |
| ENTPD5 | Homo sapiens | ectonucleoside triphosphate diphosphohydrolase 5(ENTPD5) |
| ENTPD8 | Homo sapiens | ectonucleoside triphosphate diphosphohydrolase 8(ENTPD8) |
| EPHX1 | Homo sapiens | epoxide hydrolase 1(EPHX1) |
| EPHX2 | Homo sapiens | epoxide hydrolase 2(EPHX2) |
| EPRS | Homo sapiens | glutamyl-prolyl-tRNA synthetase(EPRS) |
| ETNK1 | Homo sapiens | ethanolamine kinase 1(ETNK1) |
| ETNK2 | Homo sapiens | ethanolamine kinase 2(ETNK2) |
| FADS2 | Homo sapiens | fatty acid desaturase 2(FADS2) |
| FAH | Homo sapiens | fumarylacetoacetate hydrolase(FAH) |
| FBP1 | Homo sapiens | fructose-bisphosphatase 1(FBP1) |
| FECH | Homo sapiens | ferrochelatase(FECH) |
| FHIT | Homo sapiens | fragile histidine triad(FHIT) |
| FLAD1 | Homo sapiens | flavin adenine dinucleotide synthetase 1(FLAD1) |
| FMO1 | Homo sapiens | flavin containing monooxygenase 1(FMO1) |
| FMO2 | Homo sapiens | flavin containing monooxygenase 2(FMO2) |
| FMO3 | Homo sapiens | flavin containing monooxygenase 3(FMO3) |
| FMO5 | Homo sapiens | flavin containing monooxygenase 5(FMO5) |
| FPGT | Homo sapiens | fucose-1-phosphate guanylyltransferase(FPGT) |
| FTCD | Homo sapiens | formimidoyltransferase cyclodeaminase(FTCD) |
| FTH1 | Homo sapiens | ferritin heavy chain 1(FTH1) |
| FTMT | Homo sapiens | ferritin mitochondrial(FTMT) |
| G6PC | Homo sapiens | glucose-6-phosphatase catalytic subunit(G6PC) |
| G6PC2 | Homo sapiens | glucose-6-phosphatase catalytic subunit 2(G6PC2) |
| G6PD | Homo sapiens | glucose-6-phosphate dehydrogenase(G6PD) |
| GAA | Homo sapiens | glucosidase alpha, acid(GAA) |
| GAD1 | Homo sapiens | glutamate decarboxylase 1(GAD1) |
| GAD2 | Homo sapiens | glutamate decarboxylase 2(GAD2) |
| GAL3ST1 | Homo sapiens | galactose-3-O-sulfotransferase 1(GAL3ST1) |
| GALC | Homo sapiens | galactosylceramidase(GALC) |
| GALE | Homo sapiens | UDP-galactose-4-epimerase(GALE) |
| GALK1 | Homo sapiens | galactokinase 1(GALK1) |
| GALK2 | Homo sapiens | galactokinase 2(GALK2) |
| GALT | Homo sapiens | galactose-1-phosphate uridylyltransferase(GALT) |
| GAMT | Homo sapiens | guanidinoacetate N-methyltransferase(GAMT) |
| GANC | Homo sapiens | glucosidase alpha, neutral C(GANC) |
| GART | Homo sapiens | phosphoribosylglycinamide formyltransferase, phosphoribosylglycinamide synthetase, phosphoribosylaminoimidazole synthetase(GART) |
| GATM | Homo sapiens | glycine amidinotransferase(GATM) |
| GBE1 | Homo sapiens | 1,4-alpha-glucan branching enzyme 1(GBE1) |
| GCAT | Homo sapiens | glycine C-acetyltransferase(GCAT) |
| GCDH | Homo sapiens | glutaryl-CoA dehydrogenase(GCDH) |
| GCK | Homo sapiens | glucokinase(GCK) |
| GCLC | Homo sapiens | glutamate-cysteine ligase catalytic subunit(GCLC) |
| GCLM | Homo sapiens | glutamate-cysteine ligase modifier subunit(GCLM) |
| GDA | Homo sapiens | guanine deaminase(GDA) |
| Gene | Species | Gene Name |
| GFPT1 | Homo sapiens | glutamine--fructose-6-phosphate transaminase 1(GFPT1) |
| GFPT2 | Homo sapiens | glutamine-fructose-6-phosphate transaminase 2(GFPT2) |
| GGCT | Homo sapiens | gamma-glutamylcyclotransferase(GGCT) |
| GGT1 | Homo sapiens | gamma-glutamyltransferase 1(GGT1) |
| GGT5 | Homo sapiens | gamma-glutamyltransferase 5(GGT5) |
| GGT6 | Homo sapiens | gamma-glutamyltransferase 6(GGT6) |
| GGT7 | Homo sapiens | gamma-glutamyltransferase 7(GGT7) |
| GK | Homo sapiens | glycerol kinase(GK) |
| GK2 | Homo sapiens | glycerol kinase 2(GK2) |
| GLA | Homo sapiens | galactosidase alpha(GLA) |
| GLB1 | Homo sapiens | galactosidase beta 1(GLB1) |
| GLDC | Homo sapiens | glycine decarboxylase(GLDC) |
| GLO1 | Homo sapiens | glyoxalase I(GLO1) |
| GLS | Homo sapiens | glutaminase(GLS) |
| GLS2 | Homo sapiens | glutaminase 2(GLS2) |
| GLUD1 | Homo sapiens | glutamate dehydrogenase 1(GLUD1) |
| GLUD2 | Homo sapiens | glutamate dehydrogenase 2(GLUD2) |
| GLUL | Homo sapiens | glutamate-ammonia ligase(GLUL) |
| GLYCTK | Homo sapiens | glycerate kinase(GLYCTK) |
| GMDS | Homo sapiens | GDP-mannose 4,6-dehydratase(GMDS) |
| GMPPA | Homo sapiens | GDP-mannose pyrophosphorylase A(GMPPA) |
| GMPPB | Homo sapiens | GDP-mannose pyrophosphorylase B(GMPPB) |
| GMPR | Homo sapiens | guanosine monophosphate reductase(GMPR) |
| GMPR2 | Homo sapiens | guanosine monophosphate reductase 2(GMPR2) |
| GMPS | Homo sapiens | guanine monophosphate synthase(GMPS) |
| GNE | Homo sapiens | glucosamine (UDP-N-acetyl)-2-epimerase/N-acetylmannosamine kinase(GNE) |
| GNMT | Homo sapiens | glycine N-methyltransferase(GNMT) |
| GNPAT | Homo sapiens | glyceronephosphate O-acyltransferase(GNPAT) |
| GNPDA1 | Homo sapiens | glucosamine-6-phosphate deaminase 1(GNPDA1) |
| GNPDA2 | Homo sapiens | glucosamine-6-phosphate deaminase 2(GNPDA2) |
| GOT1 | Homo sapiens | glutamic-oxaloacetic transaminase 1(GOT1) |
| GOT2 | Homo sapiens | glutamic-oxaloacetic transaminase 2(GOT2) |
| GPAM | Homo sapiens | glycerol-3-phosphate acyltransferase, mitochondrial(GPAM) |
| GPAT2 | Homo sapiens | glycerol-3-phosphate acyltransferase 2, mitochondrial(GPAT2) |
| GPAT3 | Homo sapiens | glycerol-3-phosphate acyltransferase 3(GPAT3) |
| GPAT4 | Homo sapiens | glycerol-3-phosphate acyltransferase 4(GPAT4) |
| GPD1 | Homo sapiens | glycerol-3-phosphate dehydrogenase 1(GPD1) |
| GPD1L | Homo sapiens | glycerol-3-phosphate dehydrogenase 1-like(GPD1L) |
| GPD2 | Homo sapiens | glycerol-3-phosphate dehydrogenase 2(GPD2) |
| GPI | Homo sapiens | glucose-6-phosphate isomerase(GPI) |
| GPT | Homo sapiens | glutamic--pyruvic transaminase(GPT) |
| GPT2 | Homo sapiens | glutamic--pyruvic transaminase 2(GPT2) |
| GPX2 | Homo sapiens | glutathione peroxidase 2(GPX2) |
| GPX3 | Homo sapiens | glutathione peroxidase 3(GPX3) |
| GPX4 | Homo sapiens | glutathione peroxidase 4(GPX4) |
| GPX5 | Homo sapiens | glutathione peroxidase 5(GPX5) |
| GPX6 | Homo sapiens | glutathione peroxidase 6(GPX6) |
| GPX7 | Homo sapiens | glutathione peroxidase 7(GPX7) |
| GSR | Homo sapiens | glutathione-disulfide reductase(GSR) |
| GSS | Homo sapiens | glutathione synthetase(GSS) |
| GSTA1 | Homo sapiens | glutathione S-transferase alpha 1(GSTA1) |
| GSTA3 | Homo sapiens | glutathione S-transferase alpha 3(GSTA3) |
| GSTA4 | Homo sapiens | glutathione S-transferase alpha 4(GSTA4) |
| GSTA5 | Homo sapiens | glutathione S-transferase alpha 5(GSTA5) |
| GSTK1 | Homo sapiens | glutathione S-transferase kappa 1(GSTK1) |
| GSTM1 | Homo sapiens | glutathione S-transferase mu 1(GSTM1) |
| GSTM2 | Homo sapiens | glutathione S-transferase mu 2(GSTM2) |
| GSTM3 | Homo sapiens | glutathione S-transferase mu 3(GSTM3) |
| GSTM4 | Homo sapiens | glutathione S-transferase mu 4(GSTM4) |
| GSTM5 | Homo sapiens | glutathione S-transferase mu 5(GSTM5) |
| GSTO1 | Homo sapiens | glutathione S-transferase omega 1(GSTO1) |
| GSTO2 | Homo sapiens | glutathione S-transferase omega 2(GSTO2) |
| GSTP1 | Homo sapiens | glutathione S-transferase pi 1(GSTP1) |
| GSTZ1 | Homo sapiens | glutathione S-transferase zeta 1(GSTZ1) |
| GUCY1A1 | Homo sapiens | guanylate cyclase 1 soluble subunit alpha 1(GUCY1A1) |
| GUCY1A2 | Homo sapiens | guanylate cyclase 1 soluble subunit alpha 2(GUCY1A2) |
| GUCY1B1 | Homo sapiens | guanylate cyclase 1 soluble subunit beta 1(GUCY1B1) |
| GUCY2C | Homo sapiens | guanylate cyclase 2C(GUCY2C) |
| GUCY2D | Homo sapiens | guanylate cyclase 2D, retinal(GUCY2D) |
| GUCY2F | Homo sapiens | guanylate cyclase 2F, retinal(GUCY2F) |
| GUK1 | Homo sapiens | guanylate kinase 1(GUK1) |
| GUSB | Homo sapiens | glucuronidase beta(GUSB) |
| GYS1 | Homo sapiens | glycogen synthase 1(GYS1) |
| HAAO | Homo sapiens | 3-hydroxyanthranilate 3,4-dioxygenase(HAAO) |
| HADH | Homo sapiens | hydroxyacyl-CoA dehydrogenase(HADH) |
| HADHA | Homo sapiens | hydroxyacyl-CoA dehydrogenase/3-ketoacyl-CoA thiolase/enoyl-CoA hydratase (trifunctional protein), alpha subunit(HADHA) |
| HADHB | Homo sapiens | hydroxyacyl-CoA dehydrogenase/3-ketoacyl-CoA thiolase/enoyl-CoA hydratase (trifunctional protein), beta subunit(HADHB) |
| HAGH | Homo sapiens | hydroxyacylglutathione hydrolase(HAGH) |
| HAGHL | Homo sapiens | hydroxyacylglutathione hydrolase-like(HAGHL) |
| HAL | Homo sapiens | histidine ammonia-lyase(HAL) |
| HAO1 | Homo sapiens | hydroxyacid oxidase 1(HAO1) |
| HAO2 | Homo sapiens | hydroxyacid oxidase 2(HAO2) |
| HCCS | Homo sapiens | holocytochrome c synthase(HCCS) |
| HDC | Homo sapiens | histidine decarboxylase(HDC) |
| HEMK1 | Homo sapiens | HemK methyltransferase family member 1(HEMK1) |
| HEXA | Homo sapiens | hexosaminidase subunit alpha(HEXA) |
| HEXB | Homo sapiens | hexosaminidase subunit beta(HEXB) |
| HGD | Homo sapiens | homogentisate 1,2-dioxygenase(HGD) |
| HIBCH | Homo sapiens | 3-hydroxyisobutyryl-CoA hydrolase(HIBCH) |
| HK1 | Homo sapiens | hexokinase 1(HK1) |
| HK2 | Homo sapiens | hexokinase 2(HK2) |
| HK3 | Homo sapiens | hexokinase 3(HK3) |
| HMBS | Homo sapiens | hydroxymethylbilane synthase(HMBS) |
| HMGCL | Homo sapiens | 3-hydroxymethyl-3-methylglutaryl-CoA lyase(HMGCL) |
| HMGCS1 | Homo sapiens | 3-hydroxy-3-methylglutaryl-CoA synthase 1(HMGCS1) |
| HMGCS2 | Homo sapiens | 3-hydroxy-3-methylglutaryl-CoA synthase 2(HMGCS2) |
| HMOX1 | Homo sapiens | heme oxygenase 1(HMOX1) |
| HMOX2 | Homo sapiens | heme oxygenase 2(HMOX2) |
| HNMT | Homo sapiens | histamine N-methyltransferase(HNMT) |
| HPD | Homo sapiens | 4-hydroxyphenylpyruvate dioxygenase(HPD) |
| HPGDS | Homo sapiens | hematopoietic prostaglandin D synthase(HPGDS) |
| HPRT1 | Homo sapiens | hypoxanthine phosphoribosyltransferase 1(HPRT1) |
| HYI | Homo sapiens | hydroxypyruvate isomerase (putative)(HYI) |
| IDH2 | Homo sapiens | isocitrate dehydrogenase (NADP(+)) 2, mitochondrial(IDH2) |
| IDO1 | Homo sapiens | indoleamine 2,3-dioxygenase 1(IDO1) |
| IDO2 | Homo sapiens | indoleamine 2,3-dioxygenase 2(IDO2) |
| IL4I1 | Homo sapiens | interleukin 4 induced 1(IL4I1) |
| IMPA1 | Homo sapiens | inositol monophosphatase 1(IMPA1) |
| IMPA2 | Homo sapiens | inositol monophosphatase 2(IMPA2) |
| IMPDH1 | Homo sapiens | inosine monophosphate dehydrogenase 1(IMPDH1) |
| IMPDH2 | Homo sapiens | inosine monophosphate dehydrogenase 2(IMPDH2) |
| INMT | Homo sapiens | indolethylamine N-methyltransferase(INMT) |
| INPP1 | Homo sapiens | inositol polyphosphate-1-phosphatase(INPP1) |
| INPP4A | Homo sapiens | inositol polyphosphate-4-phosphatase type I A(INPP4A) |
| INPP4B | Homo sapiens | inositol polyphosphate-4-phosphatase type II B(INPP4B) |
| INPP5A | Homo sapiens | inositol polyphosphate-5-phosphatase A(INPP5A) |
| INPP5B | Homo sapiens | inositol polyphosphate-5-phosphatase B(INPP5B) |
| INPP5E | Homo sapiens | inositol polyphosphate-5-phosphatase E(INPP5E) |
| INPP5J | Homo sapiens | inositol polyphosphate-5-phosphatase J(INPP5J) |
| INPP5K | Homo sapiens | inositol polyphosphate-5-phosphatase K(INPP5K) |
| INPPL1 | Homo sapiens | inositol polyphosphate phosphatase like 1(INPPL1) |
| IPMK | Homo sapiens | inositol polyphosphate multikinase(IPMK) |
| IPPK | Homo sapiens | inositol-pentakisphosphate 2-kinase(IPPK) |
| ISYNA1 | Homo sapiens | inositol-3-phosphate synthase 1(ISYNA1) |
| ITPA | Homo sapiens | inosine triphosphatase(ITPA) |
| ITPK1 | Homo sapiens | inositol-tetrakisphosphate 1-kinase(ITPK1) |
| ITPKA | Homo sapiens | inositol-trisphosphate 3-kinase A(ITPKA) |
| ITPKB | Homo sapiens | inositol-trisphosphate 3-kinase B(ITPKB) |
| JMJD7-PLA2G4B | Homo sapiens | JMJD7-PLA2G4B readthrough(JMJD7-PLA2G4B) |
| KDSR | Homo sapiens | 3-ketodihydrosphingosine reductase(KDSR) |
| KHK | Homo sapiens | ketohexokinase(KHK) |
| KMO | Homo sapiens | kynurenine 3-monooxygenase(KMO) |
| KYNU | Homo sapiens | kynureninase(KYNU) |
| L2HGDH | Homo sapiens | L-2-hydroxyglutarate dehydrogenase(L2HGDH) |
| LALBA | Homo sapiens | lactalbumin alpha(LALBA) |
| LAP3 | Homo sapiens | leucine aminopeptidase 3(LAP3) |
| LCAT | Homo sapiens | lecithin-cholesterol acyltransferase(LCAT) |
| LCLAT1 | Homo sapiens | lysocardiolipin acyltransferase 1(LCLAT1) |
| LCMT1 | Homo sapiens | leucine carboxyl methyltransferase 1(LCMT1) |
| LCMT2 | Homo sapiens | leucine carboxyl methyltransferase 2(LCMT2) |
| LCT | Homo sapiens | lactase(LCT) |
| LDHA | Homo sapiens | lactate dehydrogenase A(LDHA) |
| LDHAL6A | Homo sapiens | lactate dehydrogenase A like 6A(LDHAL6A) |
| LDHAL6B | Homo sapiens | lactate dehydrogenase A like 6B(LDHAL6B) |
| LDHB | Homo sapiens | lactate dehydrogenase B(LDHB) |
| LDHC | Homo sapiens | lactate dehydrogenase C(LDHC) |
| LDHD | Homo sapiens | lactate dehydrogenase D(LDHD) |
| LIPC | Homo sapiens | lipase C, hepatic type(LIPC) |
| LIPF | Homo sapiens | lipase F, gastric type(LIPF) |
| LIPG | Homo sapiens | lipase G, endothelial type(LIPG) |
| LPCAT1 | Homo sapiens | lysophosphatidylcholine acyltransferase 1(LPCAT1) |
| LPCAT2 | Homo sapiens | lysophosphatidylcholine acyltransferase 2(LPCAT2) |
| LPCAT3 | Homo sapiens | lysophosphatidylcholine acyltransferase 3(LPCAT3) |
| LPCAT4 | Homo sapiens | lysophosphatidylcholine acyltransferase 4(LPCAT4) |
| LPGAT1 | Homo sapiens | lysophosphatidylglycerol acyltransferase 1(LPGAT1) |
| LPL | Homo sapiens | lipoprotein lipase(LPL) |
| LRAT | Homo sapiens | lecithin retinol acyltransferase (phosphatidylcholine--retinol O-acyltransferase)(LRAT) |
| LTA4H | Homo sapiens | leukotriene A4 hydrolase(LTA4H) |
| LTC4S | Homo sapiens | leukotriene C4 synthase(LTC4S) |
| LYPLA1 | Homo sapiens | lysophospholipase I(LYPLA1) |
| LYPLA2 | Homo sapiens | lysophospholipase II(LYPLA2) |
| MAOA | Homo sapiens | monoamine oxidase A(MAOA) |
| MAOB | Homo sapiens | monoamine oxidase B(MAOB) |
| MARS | Homo sapiens | methionyl-tRNA synthetase(MARS) |
| MAT1A | Homo sapiens | methionine adenosyltransferase 1A(MAT1A) |
| MAT2A | Homo sapiens | methionine adenosyltransferase 2A(MAT2A) |
| MAT2B | Homo sapiens | methionine adenosyltransferase 2B(MAT2B) |
| MBOAT2 | Homo sapiens | membrane bound O-acyltransferase domain containing 2(MBOAT2) |
| MBOAT7 | Homo sapiens | membrane bound O-acyltransferase domain containing 7(MBOAT7) |
| MCEE | Homo sapiens | methylmalonyl-CoA epimerase(MCEE) |
| MDH1 | Homo sapiens | malate dehydrogenase 1(MDH1) |
| MDH2 | Homo sapiens | malate dehydrogenase 2(MDH2) |
| ME1 | Homo sapiens | malic enzyme 1(ME1) |
| ME2 | Homo sapiens | malic enzyme 2(ME2) |
| ME3 | Homo sapiens | malic enzyme 3(ME3) |
| METTL2B | Homo sapiens | methyltransferase like 2B(METTL2B) |
| METTL6 | Homo sapiens | methyltransferase like 6(METTL6) |
| MGAM | Homo sapiens | maltase-glucoamylase(MGAM) |
| MGLL | Homo sapiens | monoglyceride lipase(MGLL) |
| MGST1 | Homo sapiens | microsomal glutathione S-transferase 1(MGST1) |
| MGST2 | Homo sapiens | microsomal glutathione S-transferase 2(MGST2) |
| MGST3 | Homo sapiens | microsomal glutathione S-transferase 3(MGST3) |
| MIF | Homo sapiens | macrophage migration inhibitory factor (glycosylation-inhibiting factor)(MIF) |
| MINPP1 | Homo sapiens | multiple inositol-polyphosphate phosphatase 1(MINPP1) |
| MIOX | Homo sapiens | myo-inositol oxygenase(MIOX) |
| MLYCD | Homo sapiens | malonyl-CoA decarboxylase(MLYCD) |
| MMAB | Homo sapiens | methylmalonic aciduria (cobalamin deficiency) cblB type(MMAB) |
| MPI | Homo sapiens | mannose phosphate isomerase(MPI) |
| MPST | Homo sapiens | mercaptopyruvate sulfurtransferase(MPST) |
| MTAP | Homo sapiens | methylthioadenosine phosphorylase(MTAP) |
| MTHFD1 | Homo sapiens | methylenetetrahydrofolate dehydrogenase, cyclohydrolase and formyltetrahydrofolate synthetase 1(MTHFD1) |
| MTHFD1L | Homo sapiens | methylenetetrahydrofolate dehydrogenase (NADP+ dependent) 1-like(MTHFD1L) |
| MTHFD2 | Homo sapiens | methylenetetrahydrofolate dehydrogenase (NADP+ dependent) 2, methenyltetrahydrofolate cyclohydrolase(MTHFD2) |
| MTHFD2L | Homo sapiens | methylenetetrahydrofolate dehydrogenase (NADP+ dependent) 2-like(MTHFD2L) |
| MTMR1 | Homo sapiens | myotubularin related protein 1(MTMR1) |
| MTMR2 | Homo sapiens | myotubularin related protein 2(MTMR2) |
| MTMR6 | Homo sapiens | myotubularin related protein 6(MTMR6) |
| MTMR7 | Homo sapiens | myotubularin related protein 7(MTMR7) |
| MTR | Homo sapiens | 5-methyltetrahydrofolate-homocysteine methyltransferase(MTR) |
| NAA80 | Homo sapiens | N-alpha-acetyltransferase 80(NAA80) |
| NADK | Homo sapiens | NAD kinase(NADK) |
| NADSYN1 | Homo sapiens | NAD synthetase 1(NADSYN1) |
| NAGK | Homo sapiens | N-acetylglucosamine kinase(NAGK) |
| NAGS | Homo sapiens | N-acetylglutamate synthase(NAGS) |
| NAMPT | Homo sapiens | nicotinamide phosphoribosyltransferase(NAMPT) |
| NANP | Homo sapiens | N-acetylneuraminic acid phosphatase(NANP) |
| NANS | Homo sapiens | N-acetylneuraminate synthase(NANS) |
| NAT1 | Homo sapiens | N-acetyltransferase 1(NAT1) |
| NAT2 | Homo sapiens | N-acetyltransferase 2(NAT2) |
| NEU1 | Homo sapiens | neuraminidase 1(NEU1) |
| NEU2 | Homo sapiens | neuraminidase 2(NEU2) |
| NEU3 | Homo sapiens | neuraminidase 3(NEU3) |
| NEU4 | Homo sapiens | neuraminidase 4(NEU4) |
| NIT2 | Homo sapiens | nitrilase family member 2(NIT2) |
| NME1 | Homo sapiens | NME/NM23 nucleoside diphosphate kinase 1(NME1) |
| NME1-NME2 | Homo sapiens | NME1-NME2 readthrough(NME1-NME2) |
| NME3 | Homo sapiens | NME/NM23 nucleoside diphosphate kinase 3(NME3) |
| NME4 | Homo sapiens | NME/NM23 nucleoside diphosphate kinase 4(NME4) |
| NME5 | Homo sapiens | NME/NM23 family member 5(NME5) |
| NME6 | Homo sapiens | NME/NM23 nucleoside diphosphate kinase 6(NME6) |
| NME7 | Homo sapiens | NME/NM23 family member 7(NME7) |
| NMNAT1 | Homo sapiens | nicotinamide nucleotide adenylyltransferase 1(NMNAT1) |
| NMNAT2 | Homo sapiens | nicotinamide nucleotide adenylyltransferase 2(NMNAT2) |
| NMNAT3 | Homo sapiens | nicotinamide nucleotide adenylyltransferase 3(NMNAT3) |
| NMRK1 | Homo sapiens | nicotinamide riboside kinase 1(NMRK1) |
| NNMT | Homo sapiens | nicotinamide N-methyltransferase(NNMT) |
| NNT | Homo sapiens | nicotinamide nucleotide transhydrogenase(NNT) |
| NOS1 | Homo sapiens | nitric oxide synthase 1(NOS1) |
| NOS2 | Homo sapiens | nitric oxide synthase 2(NOS2) |
| NOS3 | Homo sapiens | nitric oxide synthase 3(NOS3) |
| NPL | Homo sapiens | N-acetylneuraminate pyruvate lyase(NPL) |
| NPR1 | Homo sapiens | natriuretic peptide receptor 1(NPR1) |
| NPR2 | Homo sapiens | natriuretic peptide receptor 2(NPR2) |
| NT5C | Homo sapiens | 5', 3'-nucleotidase, cytosolic(NT5C) |
| NT5C1A | Homo sapiens | 5'-nucleotidase, cytosolic IA(NT5C1A) |
| NT5C1B | Homo sapiens | 5'-nucleotidase, cytosolic IB(NT5C1B) |
| NT5C2 | Homo sapiens | 5'-nucleotidase, cytosolic II(NT5C2) |
| NT5C3A | Homo sapiens | 5'-nucleotidase, cytosolic IIIA(NT5C3A) |
| NT5E | Homo sapiens | 5'-nucleotidase ecto(NT5E) |
| NT5M | Homo sapiens | 5',3'-nucleotidase, mitochondrial(NT5M) |
| NUDT12 | Homo sapiens | nudix hydrolase 12(NUDT12) |
| NUDT2 | Homo sapiens | nudix hydrolase 2(NUDT2) |
| NUDT5 | Homo sapiens | nudix hydrolase 5(NUDT5) |
| NUDT9 | Homo sapiens | nudix hydrolase 9(NUDT9) |
| OAT | Homo sapiens | ornithine aminotransferase(OAT) |
| OCRL | Homo sapiens | OCRL, inositol polyphosphate-5-phosphatase(OCRL) |
| ODC1 | Homo sapiens | ornithine decarboxylase 1(ODC1) |
| OGDH | Homo sapiens | oxoglutarate dehydrogenase(OGDH) |
| OGDHL | Homo sapiens | oxoglutarate dehydrogenase-like(OGDHL) |
| OTC | Homo sapiens | ornithine carbamoyltransferase(OTC) |
| OXCT1 | Homo sapiens | 3-oxoacid CoA-transferase 1(OXCT1) |
| OXCT2 | Homo sapiens | 3-oxoacid CoA-transferase 2(OXCT2) |
| P4HA1 | Homo sapiens | prolyl 4-hydroxylase subunit alpha 1(P4HA1) |
| P4HA2 | Homo sapiens | prolyl 4-hydroxylase subunit alpha 2(P4HA2) |
| P4HA3 | Homo sapiens | prolyl 4-hydroxylase subunit alpha 3(P4HA3) |
| PAFAH1B1 | Homo sapiens | platelet activating factor acetylhydrolase 1b regulatory subunit 1(PAFAH1B1) |
| PAFAH1B2 | Homo sapiens | platelet activating factor acetylhydrolase 1b catalytic subunit 2(PAFAH1B2) |
| PAFAH1B3 | Homo sapiens | platelet activating factor acetylhydrolase 1b catalytic subunit 3(PAFAH1B3) |
| PAFAH2 | Homo sapiens | platelet activating factor acetylhydrolase 2(PAFAH2) |
| PAH | Homo sapiens | phenylalanine hydroxylase(PAH) |
| PAICS | Homo sapiens | phosphoribosylaminoimidazole carboxylase; phosphoribosylaminoimidazolesuccinocarboxamide synthase(PAICS) |
| PAPSS1 | Homo sapiens | 3'-phosphoadenosine 5'-phosphosulfate synthase 1(PAPSS1) |
| PAPSS2 | Homo sapiens | 3'-phosphoadenosine 5'-phosphosulfate synthase 2(PAPSS2) |
| PC | Homo sapiens | pyruvate carboxylase(PC) |
| PCCA | Homo sapiens | propionyl-CoA carboxylase alpha subunit(PCCA) |
| PCCB | Homo sapiens | propionyl-CoA carboxylase beta subunit(PCCB) |
| PCK1 | Homo sapiens | phosphoenolpyruvate carboxykinase 1(PCK1) |
| PCK2 | Homo sapiens | phosphoenolpyruvate carboxykinase 2, mitochondrial(PCK2) |
| PCYT1A | Homo sapiens | phosphate cytidylyltransferase 1, choline, alpha(PCYT1A) |
| PCYT1B | Homo sapiens | phosphate cytidylyltransferase 1, choline, beta(PCYT1B) |
| PCYT2 | Homo sapiens | phosphate cytidylyltransferase 2, ethanolamine(PCYT2) |
| PDE10A | Homo sapiens | phosphodiesterase 10A(PDE10A) |
| PDE11A | Homo sapiens | phosphodiesterase 11A(PDE11A) |
| PDE1A | Homo sapiens | phosphodiesterase 1A(PDE1A) |
| PDE1B | Homo sapiens | phosphodiesterase 1B(PDE1B) |
| PDE1C | Homo sapiens | phosphodiesterase 1C(PDE1C) |
| PDE2A | Homo sapiens | phosphodiesterase 2A(PDE2A) |
| PDE3A | Homo sapiens | phosphodiesterase 3A(PDE3A) |
| PDE3B | Homo sapiens | phosphodiesterase 3B(PDE3B) |
| PDE4A | Homo sapiens | phosphodiesterase 4A(PDE4A) |
| PDE4B | Homo sapiens | phosphodiesterase 4B(PDE4B) |
| PDE4D | Homo sapiens | phosphodiesterase 4D(PDE4D) |
| PDE5A | Homo sapiens | phosphodiesterase 5A(PDE5A) |
| PDE6A | Homo sapiens | phosphodiesterase 6A(PDE6A) |
| PDE6B | Homo sapiens | phosphodiesterase 6B(PDE6B) |
| PDE6C | Homo sapiens | phosphodiesterase 6C(PDE6C) |
| PDE6D | Homo sapiens | phosphodiesterase 6D(PDE6D) |
| PDE7A | Homo sapiens | phosphodiesterase 7A(PDE7A) |
| PDE7B | Homo sapiens | phosphodiesterase 7B(PDE7B) |
| PDE8A | Homo sapiens | phosphodiesterase 8A(PDE8A) |
| PDE8B | Homo sapiens | phosphodiesterase 8B(PDE8B) |
| PDE9A | Homo sapiens | phosphodiesterase 9A(PDE9A) |
| PDHA1 | Homo sapiens | pyruvate dehydrogenase (lipoamide) alpha 1(PDHA1) |
| PDHA2 | Homo sapiens | pyruvate dehydrogenase (lipoamide) alpha 2(PDHA2) |
| PDHB | Homo sapiens | pyruvate dehydrogenase (lipoamide) beta(PDHB) |
| PEMT | Homo sapiens | phosphatidylethanolamine N-methyltransferase(PEMT) |
| PFAS | Homo sapiens | phosphoribosylformylglycinamidine synthase(PFAS) |
| PFKFB1 | Homo sapiens | 6-phosphofructo-2-kinase/fructose-2,6-biphosphatase 1(PFKFB1) |
| PFKFB2 | Homo sapiens | 6-phosphofructo-2-kinase/fructose-2,6-biphosphatase 2(PFKFB2) |
| PFKFB3 | Homo sapiens | 6-phosphofructo-2-kinase/fructose-2,6-biphosphatase 3(PFKFB3) |
| PFKFB4 | Homo sapiens | 6-phosphofructo-2-kinase/fructose-2,6-biphosphatase 4(PFKFB4) |
| PFKL | Homo sapiens | phosphofructokinase, liver type(PFKL) |
| PFKM | Homo sapiens | phosphofructokinase, muscle(PFKM) |
| PFKP | Homo sapiens | phosphofructokinase, platelet(PFKP) |
| PGD | Homo sapiens | phosphogluconate dehydrogenase(PGD) |
| PGM1 | Homo sapiens | phosphoglucomutase 1(PGM1) |
| PGM2 | Homo sapiens | phosphoglucomutase 2(PGM2) |
| PGM3 | Homo sapiens | phosphoglucomutase 3(PGM3) |
| PGP | Homo sapiens | phosphoglycolate phosphatase(PGP) |
| PGS1 | Homo sapiens | phosphatidylglycerophosphate synthase 1(PGS1) |
| PHGDH | Homo sapiens | phosphoglycerate dehydrogenase(PHGDH) |
| PHOSPHO1 | Homo sapiens | phosphoethanolamine/phosphocholine phosphatase(PHOSPHO1) |
| PHPT1 | Homo sapiens | phosphohistidine phosphatase 1(PHPT1) |
| PI4KA | Homo sapiens | phosphatidylinositol 4-kinase alpha(PI4KA) |
| PI4KB | Homo sapiens | phosphatidylinositol 4-kinase beta(PI4KB) |
| PIK3C2A | Homo sapiens | phosphatidylinositol-4-phosphate 3-kinase catalytic subunit type 2 alpha(PIK3C2A) |
| PIK3C2B | Homo sapiens | phosphatidylinositol-4-phosphate 3-kinase catalytic subunit type 2 beta(PIK3C2B) |
| PIK3C2G | Homo sapiens | phosphatidylinositol-4-phosphate 3-kinase catalytic subunit type 2 gamma(PIK3C2G) |
| PIK3C3 | Homo sapiens | phosphatidylinositol 3-kinase catalytic subunit type 3(PIK3C3) |
| PIK3CA | Homo sapiens | phosphatidylinositol-4,5-bisphosphate 3-kinase catalytic subunit alpha(PIK3CA) |
| PIK3CB | Homo sapiens | phosphatidylinositol-4,5-bisphosphate 3-kinase catalytic subunit beta(PIK3CB) |
| PIK3CD | Homo sapiens | phosphatidylinositol-4,5-bisphosphate 3-kinase catalytic subunit delta(PIK3CD) |
| PIK3CG | Homo sapiens | phosphatidylinositol-4,5-bisphosphate 3-kinase catalytic subunit gamma(PIK3CG) |
| PIKFYVE | Homo sapiens | phosphoinositide kinase, FYVE-type zinc finger containing(PIKFYVE) |
| PIP4K2A | Homo sapiens | phosphatidylinositol-5-phosphate 4-kinase type 2 alpha(PIP4K2A) |
| PIP4K2B | Homo sapiens | phosphatidylinositol-5-phosphate 4-kinase type 2 beta(PIP4K2B) |
| PIP4K2C | Homo sapiens | phosphatidylinositol-5-phosphate 4-kinase type 2 gamma(PIP4K2C) |
| PIP5K1A | Homo sapiens | phosphatidylinositol-4-phosphate 5-kinase type 1 alpha(PIP5K1A) |
| PIP5K1B | Homo sapiens | phosphatidylinositol-4-phosphate 5-kinase type 1 beta(PIP5K1B) |
| PIP5K1C | Homo sapiens | phosphatidylinositol-4-phosphate 5-kinase type 1 gamma(PIP5K1C) |
| PIPOX | Homo sapiens | pipecolic acid and sarcosine oxidase(PIPOX) |
| PISD | Homo sapiens | phosphatidylserine decarboxylase(PISD) |
| PKLR | Homo sapiens | pyruvate kinase, liver and RBC(PKLR) |
| PKM | Homo sapiens | pyruvate kinase, muscle(PKM) |
| PLA2G10 | Homo sapiens | phospholipase A2 group X(PLA2G10) |
| PLA2G12A | Homo sapiens | phospholipase A2 group XIIA(PLA2G12A) |
| PLA2G12B | Homo sapiens | phospholipase A2 group XIIB(PLA2G12B) |
| PLA2G15 | Homo sapiens | phospholipase A2 group XV(PLA2G15) |
| PLA2G1B | Homo sapiens | phospholipase A2 group IB(PLA2G1B) |
| PLA2G2A | Homo sapiens | phospholipase A2 group IIA(PLA2G2A) |
| PLA2G2C | Homo sapiens | phospholipase A2 group IIC(PLA2G2C) |
| PLA2G2D | Homo sapiens | phospholipase A2 group IID(PLA2G2D) |
| PLA2G2E | Homo sapiens | phospholipase A2 group IIE(PLA2G2E) |
| PLA2G2F | Homo sapiens | phospholipase A2 group IIF(PLA2G2F) |
| PLA2G3 | Homo sapiens | phospholipase A2 group III(PLA2G3) |
| PLA2G4A | Homo sapiens | phospholipase A2 group IVA(PLA2G4A) |
| PLA2G4E | Homo sapiens | phospholipase A2 group IVE(PLA2G4E) |
| PLA2G5 | Homo sapiens | phospholipase A2 group V(PLA2G5) |
| PLA2G6 | Homo sapiens | phospholipase A2 group VI(PLA2G6) |
| PLA2G7 | Homo sapiens | phospholipase A2 group VII(PLA2G7) |
| PLCB1 | Homo sapiens | phospholipase C beta 1(PLCB1) |
| PLCB2 | Homo sapiens | phospholipase C beta 2(PLCB2) |
| PLCB3 | Homo sapiens | phospholipase C beta 3(PLCB3) |
| PLCB4 | Homo sapiens | phospholipase C beta 4(PLCB4) |
| PLCD1 | Homo sapiens | phospholipase C delta 1(PLCD1) |
| PLCD3 | Homo sapiens | phospholipase C delta 3(PLCD3) |
| PLCD4 | Homo sapiens | phospholipase C delta 4(PLCD4) |
| PLCE1 | Homo sapiens | phospholipase C epsilon 1(PLCE1) |
| PLCG1 | Homo sapiens | phospholipase C gamma 1(PLCG1) |
| PLCG2 | Homo sapiens | phospholipase C gamma 2(PLCG2) |
| PLCZ1 | Homo sapiens | phospholipase C zeta 1(PLCZ1) |
| PLD1 | Homo sapiens | phospholipase D1(PLD1) |
| PLD2 | Homo sapiens | phospholipase D2(PLD2) |
| PLPP1 | Homo sapiens | phospholipid phosphatase 1(PLPP1) |
| PLPP2 | Homo sapiens | phospholipid phosphatase 2(PLPP2) |
| PLPP3 | Homo sapiens | phospholipid phosphatase 3(PLPP3) |
| PMM1 | Homo sapiens | phosphomannomutase 1(PMM1) |
| PNLIP | Homo sapiens | pancreatic lipase(PNLIP) |
| PNLIPRP1 | Homo sapiens | pancreatic lipase related protein 1(PNLIPRP1) |
| PNMT | Homo sapiens | phenylethanolamine N-methyltransferase(PNMT) |
| PNP | Homo sapiens | purine nucleoside phosphorylase(PNP) |
| PNPLA3 | Homo sapiens | patatin like phospholipase domain containing 3(PNPLA3) |
| PNPLA4 | Homo sapiens | patatin like phospholipase domain containing 4(PNPLA4) |
| PNPT1 | Homo sapiens | polyribonucleotide nucleotidyltransferase 1(PNPT1) |
| POLA1 | Homo sapiens | DNA polymerase alpha 1, catalytic subunit(POLA1) |
| POLA2 | Homo sapiens | DNA polymerase alpha 2, accessory subunit(POLA2) |
| POLD1 | Homo sapiens | DNA polymerase delta 1, catalytic subunit(POLD1) |
| POLD2 | Homo sapiens | DNA polymerase delta 2, accessory subunit(POLD2) |
| POLD3 | Homo sapiens | DNA polymerase delta 3, accessory subunit(POLD3) |
| POLD4 | Homo sapiens | DNA polymerase delta 4, accessory subunit(POLD4) |
| POLE | Homo sapiens | DNA polymerase epsilon, catalytic subunit(POLE) |
| POLE2 | Homo sapiens | DNA polymerase epsilon 2, accessory subunit(POLE2) |
| POLE3 | Homo sapiens | DNA polymerase epsilon 3, accessory subunit(POLE3) |
| POLE4 | Homo sapiens | DNA polymerase epsilon 4, accessory subunit(POLE4) |
| POLR1A | Homo sapiens | RNA polymerase I subunit A(POLR1A) |
| POLR1B | Homo sapiens | RNA polymerase I subunit B(POLR1B) |
| POLR1C | Homo sapiens | RNA polymerase I subunit C(POLR1C) |
| POLR1D | Homo sapiens | RNA polymerase I subunit D(POLR1D) |
| POLR1E | Homo sapiens | RNA polymerase I subunit E(POLR1E) |
| POLR2A | Homo sapiens | RNA polymerase II subunit A(POLR2A) |
| POLR2B | Homo sapiens | RNA polymerase II subunit B(POLR2B) |
| POLR2C | Homo sapiens | RNA polymerase II subunit C(POLR2C) |
| POLR2D | Homo sapiens | RNA polymerase II subunit D(POLR2D) |
| POLR2E | Homo sapiens | RNA polymerase II subunit E(POLR2E) |
| POLR2F | Homo sapiens | RNA polymerase II subunit F(POLR2F) |
| POLR2G | Homo sapiens | RNA polymerase II subunit G(POLR2G) |
| POLR2H | Homo sapiens | RNA polymerase II subunit H(POLR2H) |
| POLR2I | Homo sapiens | RNA polymerase II subunit I(POLR2I) |
| POLR2J | Homo sapiens | RNA polymerase II subunit J(POLR2J) |
| POLR2J3 | Homo sapiens | RNA polymerase II subunit J3(POLR2J3) |
| POLR3A | Homo sapiens | RNA polymerase III subunit A(POLR3A) |
| POLR3B | Homo sapiens | RNA polymerase III subunit B(POLR3B) |
| POLR3C | Homo sapiens | RNA polymerase III subunit C(POLR3C) |
| POLR3D | Homo sapiens | RNA polymerase III subunit D(POLR3D) |
| POLR3F | Homo sapiens | RNA polymerase III subunit F(POLR3F) |
| POLR3G | Homo sapiens | RNA polymerase III subunit G(POLR3G) |
| POLR3GL | Homo sapiens | RNA polymerase III subunit G like(POLR3GL) |
| POLR3H | Homo sapiens | RNA polymerase III subunit H(POLR3H) |
| POLR3K | Homo sapiens | RNA polymerase III subunit K(POLR3K) |
| PPAT | Homo sapiens | phosphoribosyl pyrophosphate amidotransferase(PPAT) |
| PPOX | Homo sapiens | protoporphyrinogen oxidase(PPOX) |
| PRDX6 | Homo sapiens | peroxiredoxin 6(PRDX6) |
| PRIM1 | Homo sapiens | primase (DNA) subunit 1(PRIM1) |
| PRIM2 | Homo sapiens | primase (DNA) subunit 2(PRIM2) |
| PRODH | Homo sapiens | proline dehydrogenase 1(PRODH) |
| PRODH2 | Homo sapiens | proline dehydrogenase 2(PRODH2) |
| PRPS1 | Homo sapiens | phosphoribosyl pyrophosphate synthetase 1(PRPS1) |
| PRPS1L1 | Homo sapiens | phosphoribosyl pyrophosphate synthetase 1-like 1(PRPS1L1) |
| PRPS2 | Homo sapiens | phosphoribosyl pyrophosphate synthetase 2(PRPS2) |
| PRUNE1 | Homo sapiens | prune exopolyphosphatase(PRUNE1) |
| PSAT1 | Homo sapiens | phosphoserine aminotransferase 1(PSAT1) |
| PSPH | Homo sapiens | phosphoserine phosphatase(PSPH) |
| PTDSS1 | Homo sapiens | phosphatidylserine synthase 1(PTDSS1) |
| PTDSS2 | Homo sapiens | phosphatidylserine synthase 2(PTDSS2) |
| PTEN | Homo sapiens | phosphatase and tensin homolog(PTEN) |
| PTGDS | Homo sapiens | prostaglandin D2 synthase(PTGDS) |
| PTGES | Homo sapiens | prostaglandin E synthase(PTGES) |
| PTGES2 | Homo sapiens | prostaglandin E synthase 2(PTGES2) |
| PTGIS | Homo sapiens | prostaglandin I2 synthase(PTGIS) |
| PTGS1 | Homo sapiens | prostaglandin-endoperoxide synthase 1(PTGS1) |
| PTGS2 | Homo sapiens | prostaglandin-endoperoxide synthase 2(PTGS2) |
| PYCR1 | Homo sapiens | pyrroline-5-carboxylate reductase 1(PYCR1) |
| PYCR2 | Homo sapiens | pyrroline-5-carboxylate reductase family member 2(PYCR2) |
| PYCR3 | Homo sapiens | pyrroline-5-carboxylate reductase 3(PYCR3) |
| PYGB | Homo sapiens | phosphorylase, glycogen; brain(PYGB) |
| PYGL | Homo sapiens | phosphorylase, glycogen, liver(PYGL) |
| PYGM | Homo sapiens | phosphorylase, glycogen, muscle(PYGM) |
| QPRT | Homo sapiens | quinolinate phosphoribosyltransferase(QPRT) |
| RDH10 | Homo sapiens | retinol dehydrogenase 10 (all-trans)(RDH10) |
| RDH11 | Homo sapiens | retinol dehydrogenase 11 (all-trans/9-cis/11-cis)(RDH11) |
| RDH12 | Homo sapiens | retinol dehydrogenase 12 (all-trans/9-cis/11-cis)(RDH12) |
| RDH16 | Homo sapiens | retinol dehydrogenase 16 (all-trans)(RDH16) |
| RDH5 | Homo sapiens | retinol dehydrogenase 5(RDH5) |
| RDH8 | Homo sapiens | retinol dehydrogenase 8 (all-trans)(RDH8) |
| RENBP | Homo sapiens | renin binding protein(RENBP) |
| RETSAT | Homo sapiens | retinol saturase(RETSAT) |
| RFK | Homo sapiens | riboflavin kinase(RFK) |
| RPE65 | Homo sapiens | RPE65, retinoid isomerohydrolase(RPE65) |
| RRM1 | Homo sapiens | ribonucleotide reductase catalytic subunit M1(RRM1) |
| RRM2 | Homo sapiens | ribonucleotide reductase regulatory subunit M2(RRM2) |
| RRM2B | Homo sapiens | ribonucleotide reductase regulatory TP53 inducible subunit M2B(RRM2B) |
| SARDH | Homo sapiens | sarcosine dehydrogenase(SARDH) |
| SAT1 | Homo sapiens | spermidine/spermine N1-acetyltransferase 1(SAT1) |
| SAT2 | Homo sapiens | spermidine/spermine N1-acetyltransferase family member 2(SAT2) |
| SCLY | Homo sapiens | selenocysteine lyase(SCLY) |
| SDS | Homo sapiens | serine dehydratase(SDS) |
| SEPHS1 | Homo sapiens | selenophosphate synthetase 1(SEPHS1) |
| SEPHS2 | Homo sapiens | selenophosphate synthetase 2(SEPHS2) |
| SGMS1 | Homo sapiens | sphingomyelin synthase 1(SGMS1) |
| SGMS2 | Homo sapiens | sphingomyelin synthase 2(SGMS2) |
| SGPL1 | Homo sapiens | sphingosine-1-phosphate lyase 1(SGPL1) |
| SGPP1 | Homo sapiens | sphingosine-1-phosphate phosphatase 1(SGPP1) |
| SGPP2 | Homo sapiens | sphingosine-1-phosphate phosphatase 2(SGPP2) |
| SHMT1 | Homo sapiens | serine hydroxymethyltransferase 1(SHMT1) |
| SHMT2 | Homo sapiens | serine hydroxymethyltransferase 2(SHMT2) |
| SI | Homo sapiens | sucrase-isomaltase(SI) |
| SMPD1 | Homo sapiens | sphingomyelin phosphodiesterase 1(SMPD1) |
| SMPD2 | Homo sapiens | sphingomyelin phosphodiesterase 2(SMPD2) |
| SMPD4 | Homo sapiens | sphingomyelin phosphodiesterase 4(SMPD4) |
| SMS | Homo sapiens | spermine synthase(SMS) |
| SORD | Homo sapiens | sorbitol dehydrogenase(SORD) |
| SPHK1 | Homo sapiens | sphingosine kinase 1(SPHK1) |
| SPHK2 | Homo sapiens | sphingosine kinase 2(SPHK2) |
| SPTLC1 | Homo sapiens | serine palmitoyltransferase long chain base subunit 1(SPTLC1) |
| SPTLC2 | Homo sapiens | serine palmitoyltransferase long chain base subunit 2(SPTLC2) |
| SRM | Homo sapiens | spermidine synthase(SRM) |
| SRR | Homo sapiens | serine racemase(SRR) |
| SUCLA2 | Homo sapiens | succinate-CoA ligase ADP-forming beta subunit(SUCLA2) |
| SUCLG1 | Homo sapiens | succinate-CoA ligase alpha subunit(SUCLG1) |
| SUCLG2 | Homo sapiens | succinate-CoA ligase GDP-forming beta subunit(SUCLG2) |
| SULT1A2 | Homo sapiens | sulfotransferase family 1A member 2(SULT1A2) |
| SULT1A3 | Homo sapiens | sulfotransferase family 1A member 3(SULT1A3) |
| SULT1A4 | Homo sapiens | sulfotransferase family 1A member 4(SULT1A4) |
| SULT1E1 | Homo sapiens | sulfotransferase family 1E member 1(SULT1E1) |
| SUOX | Homo sapiens | sulfite oxidase(SUOX) |
| SYNJ1 | Homo sapiens | synaptojanin 1(SYNJ1) |
| SYNJ2 | Homo sapiens | synaptojanin 2(SYNJ2) |
| TAZ | Homo sapiens | tafazzin(TAZ) |
| TBXAS1 | Homo sapiens | thromboxane A synthase 1(TBXAS1) |
| TDO2 | Homo sapiens | tryptophan 2,3-dioxygenase(TDO2) |
| TH | Homo sapiens | tyrosine hydroxylase(TH) |
| TK1 | Homo sapiens | thymidine kinase 1(TK1) |
| TK2 | Homo sapiens | thymidine kinase 2, mitochondrial(TK2) |
| TKFC | Homo sapiens | triokinase and FMN cyclase(TKFC) |
| TPH1 | Homo sapiens | tryptophan hydroxylase 1(TPH1) |
| TPH2 | Homo sapiens | tryptophan hydroxylase 2(TPH2) |
| TPI1 | Homo sapiens | triosephosphate isomerase 1(TPI1) |
| TPMT | Homo sapiens | thiopurine S-methyltransferase(TPMT) |
| TPO | Homo sapiens | thyroid peroxidase(TPO) |
| TRDMT1 | Homo sapiens | tRNA aspartic acid methyltransferase 1(TRDMT1) |
| TREH | Homo sapiens | trehalase(TREH) |
| TRMT11 | Homo sapiens | tRNA methyltransferase 11 homolog(TRMT11) |
| TSTA3 | Homo sapiens | tissue specific transplantation antigen P35B(TSTA3) |
| TXNDC12 | Homo sapiens | thioredoxin domain containing 12(TXNDC12) |
| TXNRD1 | Homo sapiens | thioredoxin reductase 1(TXNRD1) |
| TXNRD2 | Homo sapiens | thioredoxin reductase 2(TXNRD2) |
| TYMP | Homo sapiens | thymidine phosphorylase(TYMP) |
| TYMS | Homo sapiens | thymidylate synthetase(TYMS) |
| TYR | Homo sapiens | tyrosinase(TYR) |
| TYRP1 | Homo sapiens | tyrosinase related protein 1(TYRP1) |
| UAP1 | Homo sapiens | UDP-N-acetylglucosamine pyrophosphorylase 1(UAP1) |
| UCK1 | Homo sapiens | uridine-cytidine kinase 1(UCK1) |
| UCK2 | Homo sapiens | uridine-cytidine kinase 2(UCK2) |
| UCKL1 | Homo sapiens | uridine-cytidine kinase 1 like 1(UCKL1) |
| UGCG | Homo sapiens | UDP-glucose ceramide glucosyltransferase(UGCG) |
| UGDH | Homo sapiens | UDP-glucose 6-dehydrogenase(UGDH) |
| UGT1A10 | Homo sapiens | UDP glucuronosyltransferase family 1 member A10(UGT1A10) |
| UGT1A4 | Homo sapiens | UDP glucuronosyltransferase family 1 member A4(UGT1A4) |
| UGT2A1 | Homo sapiens | UDP glucuronosyltransferase family 2 member A1 complex locus(UGT2A1) |
| UGT2A3 | Homo sapiens | UDP glucuronosyltransferase family 2 member A3(UGT2A3) |
| UGT2B11 | Homo sapiens | UDP glucuronosyltransferase family 2 member B11(UGT2B11) |
| UGT2B15 | Homo sapiens | UDP glucuronosyltransferase family 2 member B15(UGT2B15) |
| UGT2B4 | Homo sapiens | UDP glucuronosyltransferase family 2 member B4(UGT2B4) |
| UGT2B7 | Homo sapiens | UDP glucuronosyltransferase family 2 member B7(UGT2B7) |
| UGT8 | Homo sapiens | UDP glycosyltransferase 8(UGT8) |
| UMPS | Homo sapiens | uridine monophosphate synthetase(UMPS) |
| UPB1 | Homo sapiens | beta-ureidopropionase 1(UPB1) |
| UPP1 | Homo sapiens | uridine phosphorylase 1(UPP1) |
| UPP2 | Homo sapiens | uridine phosphorylase 2(UPP2) |
| UPRT | Homo sapiens | uracil phosphoribosyltransferase homolog(UPRT) |
| URAD | Homo sapiens | ureidoimidazoline (2-oxo-4-hydroxy-4-carboxy-5-) decarboxylase(URAD) |
| UROC1 | Homo sapiens | urocanate hydratase 1(UROC1) |
| UROD | Homo sapiens | uroporphyrinogen decarboxylase(UROD) |
| UROS | Homo sapiens | uroporphyrinogen III synthase(UROS) |
| UXS1 | Homo sapiens | UDP-glucuronate decarboxylase 1(UXS1) |
| WARS | Homo sapiens | tryptophanyl-tRNA synthetase(WARS) |
| WARS2 | Homo sapiens | tryptophanyl tRNA synthetase 2, mitochondrial(WARS2) |
| XDH | Homo sapiens | xanthine dehydrogenase(XDH) |
| ZNRD1 | Homo sapiens | zinc ribbon domain containing 1(ZNRD1) |

**Supplementary Table S2. The gene primers sequence.**

| **Gene** | **Primers sequence** |
| --- | --- |
| β-Actin-F | CATGTACGTTGCTATCCAGGC |
| β-Actin-R | CTCCTTAATGTCACGCACGAT |
| CD38-F | CAACTCTGTCTTGGCGTCAGT |
| CD38-R | CCCATACACTTTGGCAGTCTACA |
| INPP5E-F | CCCATACACTTTGGCAGTCTACA |
| INPP5E-R | TGCAGACGAGTCTCCCACT |
| POLR3G-F | GAGGACGTGCTGCTTATACCT |
| POLR3G-R | CTGTTCTGCGGCATCATCGT |
